# Multiscale Regulation of Ru‐Based Catalysts for Upgrading Biomass‐Derived Levulinic Acid and Levulinates to *γ*‐Valerolactone: Mechanisms, Advances, and Challenges

**DOI:** 10.1002/advs.78374

**Published:** 2026-10-18

**Authors:** Xin Ni, Cailu Xiao, Gongbing Zhou, Jingzi Zhang

**Affiliations:** ^1^ Chongqing Key Laboratory of Green Catalysis Materials and Technology College of Chemistry and Materials Science Chongqing Normal University Chongqing China; ^2^ Qingdao Central Hospital University of Health and Rehabilitation Sciences (Qingdao Central Hospital) Qingdao Shandong China; ^3^ Research Institute of Physical Sciences in Special Environments Harbin Institute of Technology Shenzhen Guangdong China

**Keywords:** *γ*‐valerolactone, biomass upgrading, levulinates, levulinic acid, regulation strategies, Ru‐based catalysts, selective hydrogenation

## Abstract

*γ*‐Valerolactone (GVL) serves as a bridge connecting biomass‐derived levulinic acid (LA) and its esters to value‐added chemicals. Ru‐based catalysts exhibit high efficiency for LA/levulinates‐to‐GVL conversion, owing to their strong C═O activation ability, excellent GVL selectivity, and compatibility to diverse hydrogen sources such as H_2_, formic acid (FA), and alcohols. Based on an analysis of the mechanisms, thermodynamics, and kinetics of this process, the review summarizes recent advances in the multiscale regulation of Ru‐based catalysts, covering electronic structure and Lewis/Brønsted acid sites, support structure, Ru particle size and dispersion, reaction conditions and solvent microenvironments, and resistance to deactivation. By integrating the two primary reaction pathways, rate‐determining steps (RDS), and reaction orders with respect to H_2_, the adoption of these strategies is proposed to follow a streamlined workflow by 1) defining reaction system, 2) eliminating heat and mass transfer limitations, 3) identifying primary limiting factor of catalytic performances, and 4) selecting and validating optimization strategy. Additionally, a brief comparison with non‐noble metal (Ni‐, Cu‐, Co‐, and Zr‐based) catalysts further reveals the conditions that justify the higher cost of Ru. These frameworks identify key metrics for catalyst design, which are anticipated to advance the efficient conversion and utilization of biomass.

## Graphical abstract

This review summarizes recent advances in the mechanistic, thermodynamic, and kinetic studies of biomass‐derived levulinic acid/levulinates conversion to *γ*‐valerolactone, along with the corresponding multiscale regulation strategies of Ru‐based catalysts. Key challenges and future outlook are also envisioned, which are expected to advance the rational design of highly efficient catalysts for sustainable biomass upgrading.

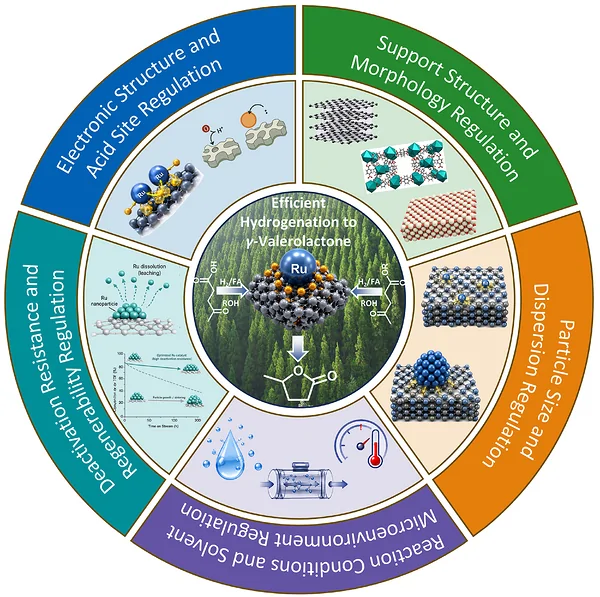

## 1 Introduction

The rapid advancement of industrial civilization has driven an ever‐increasing consumption of fossil fuels, giving rise to greenhouse gas emission, ecological degradation, and resource depletion, which now rank among the most pressing concerns [1, 2]. Developing renewable energy sources and sustainable carbon feedstocks has therefore become essential to fundamentally alleviating both the energy crisis and environmental pressures. Among the diverse renewable resources available, biomass stands out as one of the most promising alternatives to fossil fuels, owing to its wide availability, carbon‐cycle neutrality, and renewability [3]. Biomass feedstocks span agricultural and forestry residues, food and municipal organic solid wastes, and aquatic biological resources [4, 5], with lignocellulosic biomass representing the most abundant non‐edible type. Lignocellulose is composed of cellulose, hemicellulose, and lignin. This oxygen‐rich feedstock can act both as an energy carrier and as a carbon source for the chemical industry, offering a renewable alternative to petroleum [6].

Biomass valorization mainly relies on converting it into key platform molecules. These intermediates can then be catalytically upgraded into fuels and value‐added chemicals. LA ranks among the most representative biomass‐derived platform compounds, obtainable from sugars and lignocellulosic biomass via acid‐catalyzed hydrolysis and dehydration [7]. It contains both a ketone carbonyl (C═O) and a carboxyl group (─COOH). These two functional groups enable a variety of reactions, including hydrogenation, esterification, condensation, and amidation, leading to the production of chemical monomers and fuel components [7]. This chemical versatility endows LA with unique connectivity value in biorefinery networks, and the U.S. Department of Energy listed it among the top 12 biomass‐derived platform compounds in its 2004 screening report [8]. From a reaction‐network perspective, LA synthesis is tightly coupled to the cellulose conversion chain, that is, acid‐catalyzed hydrolysis of cellulose to monosaccharides, followed by transformation through intermediates such as 5‐hydroxymethylfurfural (5‐HMF), yielding LA with equimolar FA as a co‐product [9]. This co‐production feature provides a natural process‐integration basis for subsequent in situ hydrogen‐donation routes.

Among the many downstream products of LA, GVL is widely recognized as the most important hub molecule. GVL exhibits a high flash point, low vapor pressure, and excellent thermal stability at ambient pressure, combined with low toxicity and biodegradability. At the application level, GVL serves directly as a food additive, green solvent, and gasoline blending component, and can enter the chemical production system as a renewable carbon source. Upon further upgrading, it converts to high‐value products such as valeric acid, pentenyl fuel components, and polymerization monomers [10]. GVL upgrade proceeds through a multi‐pathway network. On Ru catalysts, ring‐opening hydrogenation at the C─O bond generates 1,4‐pentanediol (1,4‐PDO), which undergoes dehydration−cyclization to produce 2‐methyltetrahydrofuran (2‐MTHF), an important clean fuel blending component. GVL also undergoes ring‐opening decarbonylation to give 2‐butanol (2‐BuOH). Density functional theory (DFT) calculations indicate this pathway carries a low apparent activation energy of 61 kJ mol^−1^ on the Ru(0001) surface [11]. Under acidic conditions, ring‐opening hydrolysis of GVL affords *γ*‐valeric acid (VA), whose esterified form can be utilized directly as a renewable liquid fuel component [10]. This dual character makes GVL a bridge molecule connecting biorefinery systems with fuel and fine chemical platforms. As illustrated in Figure 1, two primary reaction pathways link lignocellulosic biomass to GVL. In the first, cellulose is hydrolyzed to glucose, isomerized and dehydrated to 5‐HMF, followed by acid‐catalyzed rehydration and C–C bond cleavage to produce LA and FA, and finally converted to GVL via a sequential hydrogenation–cyclization sequence. In the second route, hemicellulose is hydrolyzed to pentoses, dehydrated to furfural, reduced to furfuryl alcohol, and converted by alcoholysis to a levulinate ester, which subsequently undergoes hydrogenation and lactonization to GVL.

**FIGURE 1:**
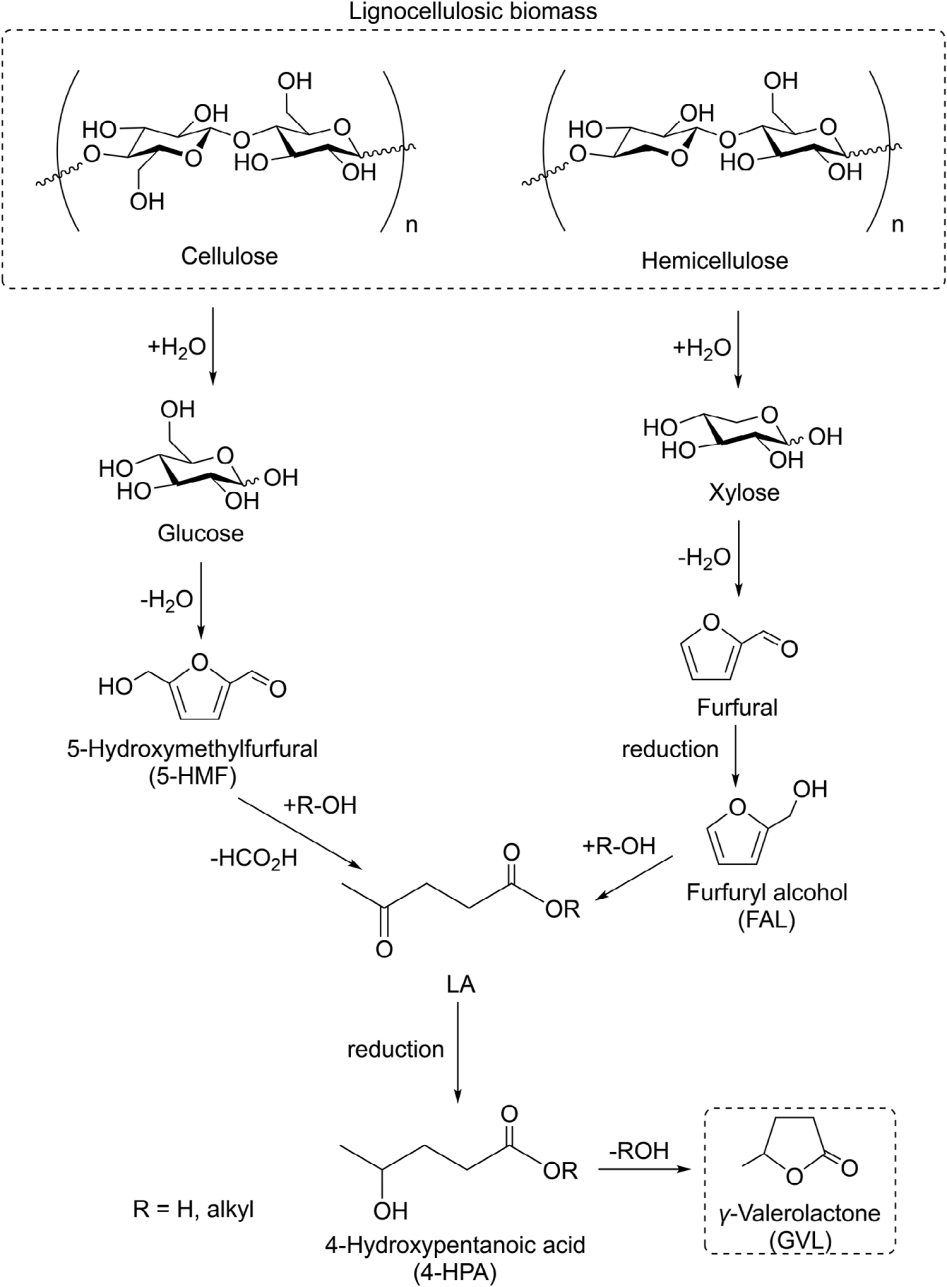
Reaction network for GVL synthesis from lignocellulosic biomass via two primary pathways. Reproduced with permission [10]. Copyright 2015, American Chemical Society.

In heterogeneous catalytic systems for selective hydrogenation of LA and its esters to GVL, an inherent trade‐off exists between hydrogenation activity and product selectivity. The strong hydrogenation capacity that drives LA conversion also promotes GVL ring‐opening and over‐hydrogenation, and high acid‐site density accelerates dehydration–cyclization while equally promoting GVL ring‐opening side reactions. Catalyst stability under hydrothermal acidic conditions remains an engineering bottleneck for scale‐up. Metal leaching, sintering, and carbon deposition are the primary deactivation pathways. Reducing dependence on externally supplied high‐pressure H_2_ is another practical issue motivating the search for alternative routes. Catalytic transfer hydrogenation (CTH) systems using FA or alcohols as hydrogen donors have therefore attracted sustained attention [12, 13]. Beyond the aforementioned CTH using the co‐produced FA as hydrogen supply [9, 12], the Meerwein−Ponndorf−Verley (MPV) reduction route using alcohols as donors offers a cost‐effective, safe alternative to high‐pressure H_2_. Besides, innovation in hydrogen‐donor strategies has advanced well beyond FA and alcohols. Liquid hydrosilane donors, exemplified by polymethylhydrosiloxane (PMHS), enable CTH of biomass‐derived carbonyl compounds over commercial Pd/C under mild conditions [14]. An oxygen‐vacancy‐rich WO_3_ semiconductor modified with a supramolecular (cucurbit[5]uril) achieves 99% GVL yield from ethyl levulinate (EL) at 25°C within 1 h under visible‐light irradiation using ammonia borane as a solid hydrogen source [15]. These advances underscore that the hydrogen‐source of LA/levulinate‐to‐GVL chemistry is far broader than conventional thermocatalytic H_2_ route.

Due to its exceptional activity for carbonyl hydrogenation and high efficiency in the dissociative adsorption of H_2_, Ru has emerged as the most widely studied catalytic system for the selective hydrogenation of LA and its esters into GVL [16, 17, 18]. Compared to noble metals such as Pt, Pd, and Ir, as well as non‐noble metals like Ni and Cu, Ru has shown high intrinsic activity and GVL selectivity in several aqueous‐phase comparisons [19]. In a systematic comparative study using activated carbon as the support and a metal loading of 5 wt%, Manzer et al. [20] found the order of catalytic activity to be Ru > Ir > Pd > Rh > Pt > Re > Ni. Notably, the 5 wt% Ru/C catalyst achieved 100% conversion of LA and over 97% selectivity for GVL. These results represent a comparison within a single study using identical supports, metal loadings, and reaction protocols, rather than a universal ranking of activity across varying catalyst structures and process conditions. Similarly, gas‐phase hydrogenation studies conducted by Sudhakar et al. confirmed that among noble metal catalysts supported on hydroxyapatite (HAP), Ru/HAP yields the highest GVL production [21]. The superior performance of Ru stems from its 4d electronic structure, which effectively activates the carbonyl oxygen in the C═O bond and significantly lowers the apparent activation energy of the hydrogenation reaction. Furthermore, Ru possesses both electrophilic and nucleophilic reactivity, thereby demonstrating exceptional capabilities for substrate adsorption and activation [22, 23, 24, 25]. These characteristics enable Ru to achieve high GVL selectivity at relatively low temperatures or within shorter reaction times. However, the catalytic function of Ru cannot be attributed solely to its electronic structure, as factors such as the number of active sites, oxidation state, metal‐support interactions, solvent effects, and the environment of acidic sites may all vary simultaneously. Therefore, mechanistic interpretations based on electronic structure or Lewis acidity must be considered in the context of specific catalysts and reaction conditions.

Although non‐noble metal catalysts such as Ni, Cu, or Fe have attracted significant interest due to their cost advantages, their overall catalytic performance and stability still struggle to fully match Ru‐based systems [26]. Their limitations include requirements for higher operating temperatures or metal loadings, as well as issues such as particle growth, changes in oxidation state, metal leaching, and support instability. Consequently, the choice of Ru should not be based solely on final conversion or yield. The preferred catalyst family is nevertheless process‐dependent. Ni‐ and Cu‐based catalysts may become attractive when higher metal loading or operating temperature is acceptable. Co‐based systems require separate consideration of heterogeneous and ligand‐based catalysts because catalyst recovery and product separation differ substantially. Zr‐based Lewis‐acid catalysts avoid noble metals and pressurized H_2_ through alcohol‐mediated transfer hydrogenation, but they require a sacrificial hydrogen donor and subsequent donor/product separation. Ru is therefore most defensible when gains in low‐temperature productivity, short‐contact‐time selectivity, catalyst lifetime, recoverability, or reduced separation demand compensate for its higher inventory cost.

Furthermore, a meta‐analysis of 150 literature datasets by Niaze et al. indicates that the ratio of Lewis acid sites (LAS) to Brønsted acid sites (BAS) (L/B ratio) is a key descriptor determining GVL selectivity. A higher L/B ratio favors the selective activation of the C═O bond in LA at LAS, thereby promoting the hydrogenation‐dehydration pathway and enhancing GVL selectivity. Conversely, excessive Brønsted acidity often leads to GVL ring‐opening and the formation of by‐products, thereby reducing selectivity [27]. However, given that the datasets encompass diverse catalyst families, measurement methods for acidic sites, and reaction conditions, the potential for confounding variables such as the nature of the support and the reaction temperature cannot be ruled out. Thus, the reported relationship should be viewed as a hypothesis‐generating correlation rather than a universally applicable causal law or a requirement specific solely to Ru‐based catalysts.

Nevertheless, the Pt‐group metal price of Ru makes improving atomic utilization both a scientific objective and an economic constraint, a point often underestimated in laboratory‐oriented studies. Resolving these issues requires cooperative design across Ru electronic structure, support interfacial microenvironment, active‐site dispersion, and operating conditions, rather than optimizing any single variable in isolation. Several comprehensive reviews on the conversion of LA and levulinates to GVL have appeared in the past 5 years. Hijazi et al. centered on FA as a hydrogen donor and the related FA decomposition chemistry [28]. Khalid et al. summarized in a CTH‐focused perspective and grouped catalysts covering zirconia‐based, zeolite, precious‐metal, and non‐precious‐metal systems [29]. Maumela et al. surveyed Ru catalysts by the effects of particle size, solvent, and support on activity and selectivity [19]. Zhang et al. classified Ru‐based catalysts by support type and metal–support interaction [30]. These reviews are valuable, and the latter two focused on Ru. Nevertheless, they are organized around hydrogen sources, catalyst classes, operating variables, or supports. In contrast, the present review begins with the reaction mechanisms and kinetic characteristics of Ru‐catalyzed conversion of LA and levulinates to GVL, and then discusses five regulation strategies including electronic structure and acid–base site regulation, support structure and morphology regulation, particle size and dispersion regulation, reaction conditions and solvent microenvironment regulation, and deactivation control and regeneration. Figure 2 illustrates the sequence for applying these strategies based on kinetic evidence derived from the reaction. First, the reaction system is characterized based on the substrate, hydrogen source, phase, reactor type, and feed composition. Next, kinetic bottlenecks are assessed and descriptors are identified to determine whether reaction rate, selectivity, or catalyst lifespan is the primary limiting factor. Based on this, one or more control strategies can be selected and evaluated experimentally. Thus, these five strategies constitute a “toolbox for optimization” based on the reaction system. This framework aims to optimize catalyst design by identifying the specific conditions required for the reaction, providing evidence of kinetic bottlenecks, and verifying the effectiveness of the control strategies. For clarity, sections 3.1–3.5 describe the five specific control strategies, while section 3.6 integrates these strategies within the defined reaction systems.

**FIGURE 2:**
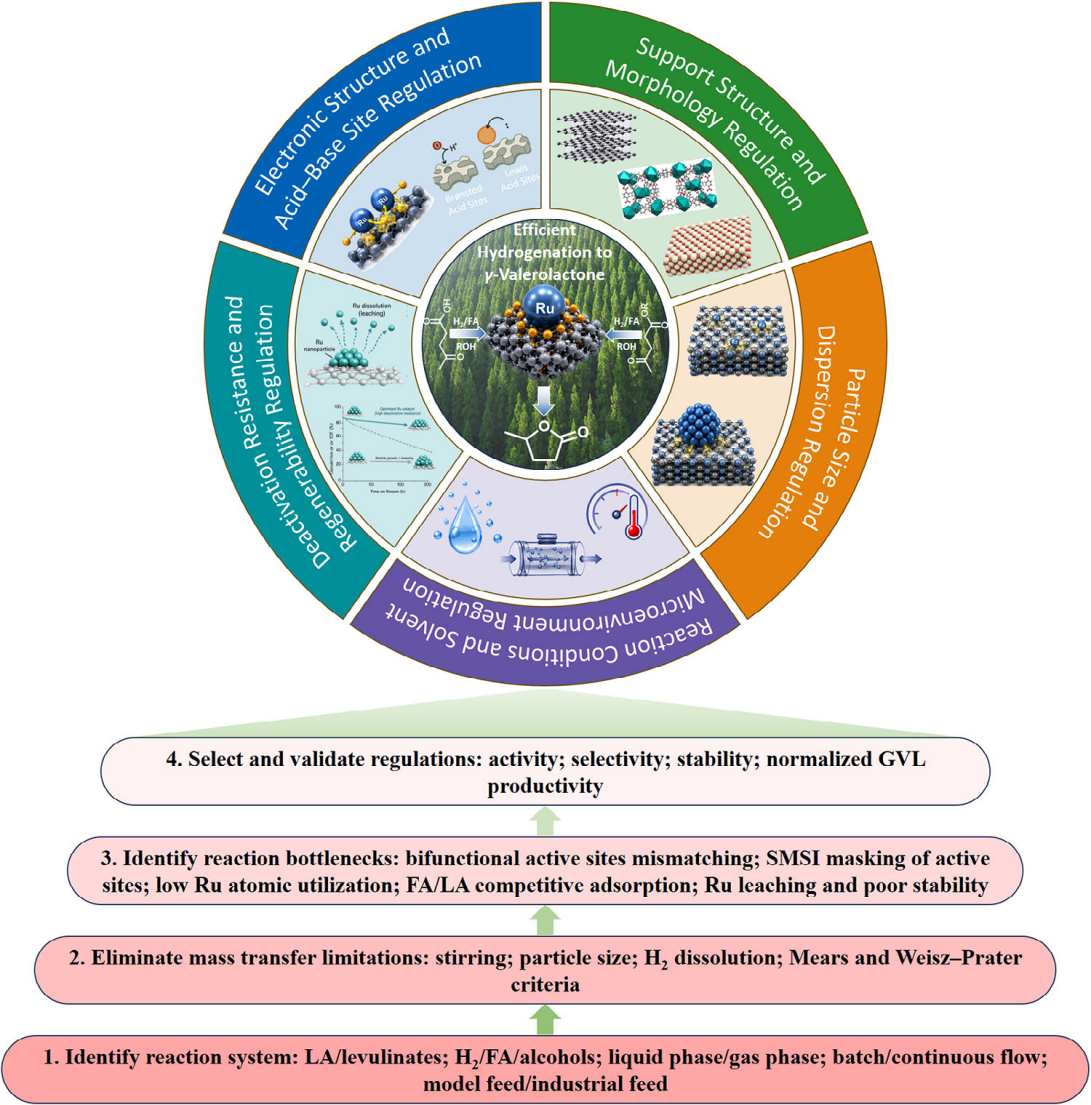
The catalyst design framework based on specific reaction systems and guided by the bottlenecks of Ru‐catalyzed LA/levulinates‐to‐GVL conversion.

## 2 Fundamentals of LA/Levulinates‐to‐GVL Conversion Over Ru‐Based Catalysts

### 2.1 Reaction Pathways and Thermodynamic Basis

Both LA and its esters are converted to GVL through a tandem hydrogenation–cyclization sequence. However, the relative order of the hydrogenation and dehydration steps differs under different reaction conditions, giving rise to two competing pathways (Figure 3) [31]. The pathway 1 passing 4‐hydroxypentanoic acid (4‐HPA) dominates under low‐temperature liquid‐phase conditions, where the LA carbonyl is first hydrogenated at metallic Ru sites to the thermodynamically unstable4‐HPA, which then undergoes intramolecular dehydration–esterification at the acid sites of catalyst to yield GVL. The pathway 2 passing *α*‐angelica lactone (*α*‐AL) prevails under high‐temperature gas‐phase conditions, where LA is first enolized and dehydrated intramolecularly at acid sites to give *α*‐AL, which isomerizes to *β*‐AL under acidic conditions and is subsequently hydrogenated selectively at Ru sites to GVL [32]. The two pathways differ fundamentally in their thermodynamic driving forces, kinetic features, and active‐site requirements. These differences directly dictate the design logic for bifunctional catalysts.

**FIGURE 3:**
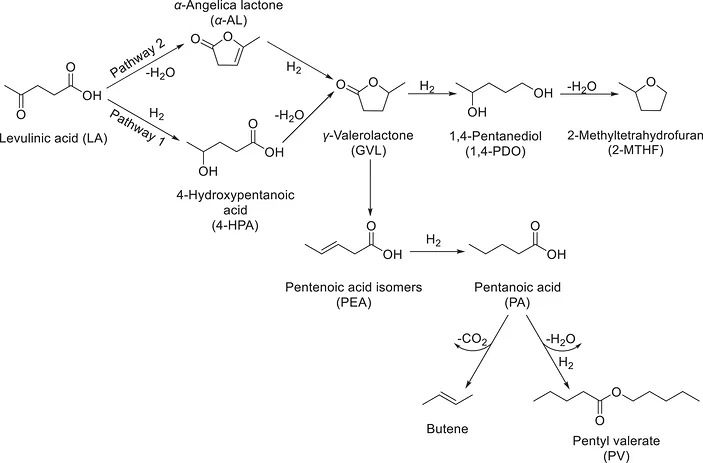
Reaction pathways of LA‐to‐GVL conversion and the downstream conversion network of GVL. Reproduced with permission [31]. Copyright 2016, Elsevier.

The pathway 1 in Figure 3 begins with heterolytic dissociation of molecular hydrogen at Ru active sites to generate reactive surface hydrogen species (H^*^). DFT calculations indicate that the H_2_ dissociation barrier on Ru nanocluster surfaces is negligibly low and does not constitute the RDS [33]. The coordination environment and electronic structure of Ru sites, however, critically regulate H^*^ formation efficiency. LA adsorption energy on Ru sites is another key factor, with a value of −0.53 eV on intact Ru(0001) decreasing to −0.95 eV at coordinatively unsaturated Ru sites [33]. This stronger adsorption is consistent with enhanced substrate interaction at coordinatively unsaturated sites, although the intrinsic activity also depends on the barriers and surface coverages of subsequent steps. Ru K‐edge X‐ray absorption near‐edge structure/extended X‐ray absorption fine structure (XANES/EXAFS) characterization confirms that under reaction conditions, Ru° constitutes the principal active form, and its degree of coordinative unsaturation correlates positively with LA hydrogenation activity [33]. Following H_2_ dissociation to H^*^, the LA carbonyl hydrogenation undergoes either by initial C─H bond formation (alkoxy pathway) or O═H bond formation (hydroxy pathway) of the ketone group. On the intact Ru(0001) surface, DFT calculations along the alkoxy pathway show that the activation energy for the alkoxy intermediate formation is 0.67 eV. The alkoxy intermediate is then further hydrogenated to 4‐HPA, with an activation energy of 1.29 eV, which constitutes the RDS of the alkoxy pathway. The competing hydroxy pathway, in which hydrogen first adds to the carbonyl oxygen, exhibits a higher first‐step barrier of 1.36 eV and is therefore disfavored (Figure 4a,b) [34]. Microkinetic modeling by Mamun et al. established that the apparent activation energy for LA hydrogenation on Ru(0001) in 1,4‐dioxane is 1.22 eV, substantially higher than 1.02 eV in the aqueous system, highlighting the pronounced effect of solvation on the energy barrier [35]. At ZnO_x_‐derived defective Ru sites, the coordinatively unsaturated configuration reduces the barriers for the addition of H^*^ on carbonyl C and carbonyl O to 0.09 and 0.29 eV, respectively, as compared to 0.22 and 0.31 eV on intact Ru, elevating activity by orders of magnitude (Figure 4c–e) [33]. These results provide the thermodynamic basis for metal–support synergistic activation of the LA carbonyl and form the theoretical foundation for the Ru electronic‐structure regulation studies discussed in Sections 3.1–3.3.

**FIGURE 4:**
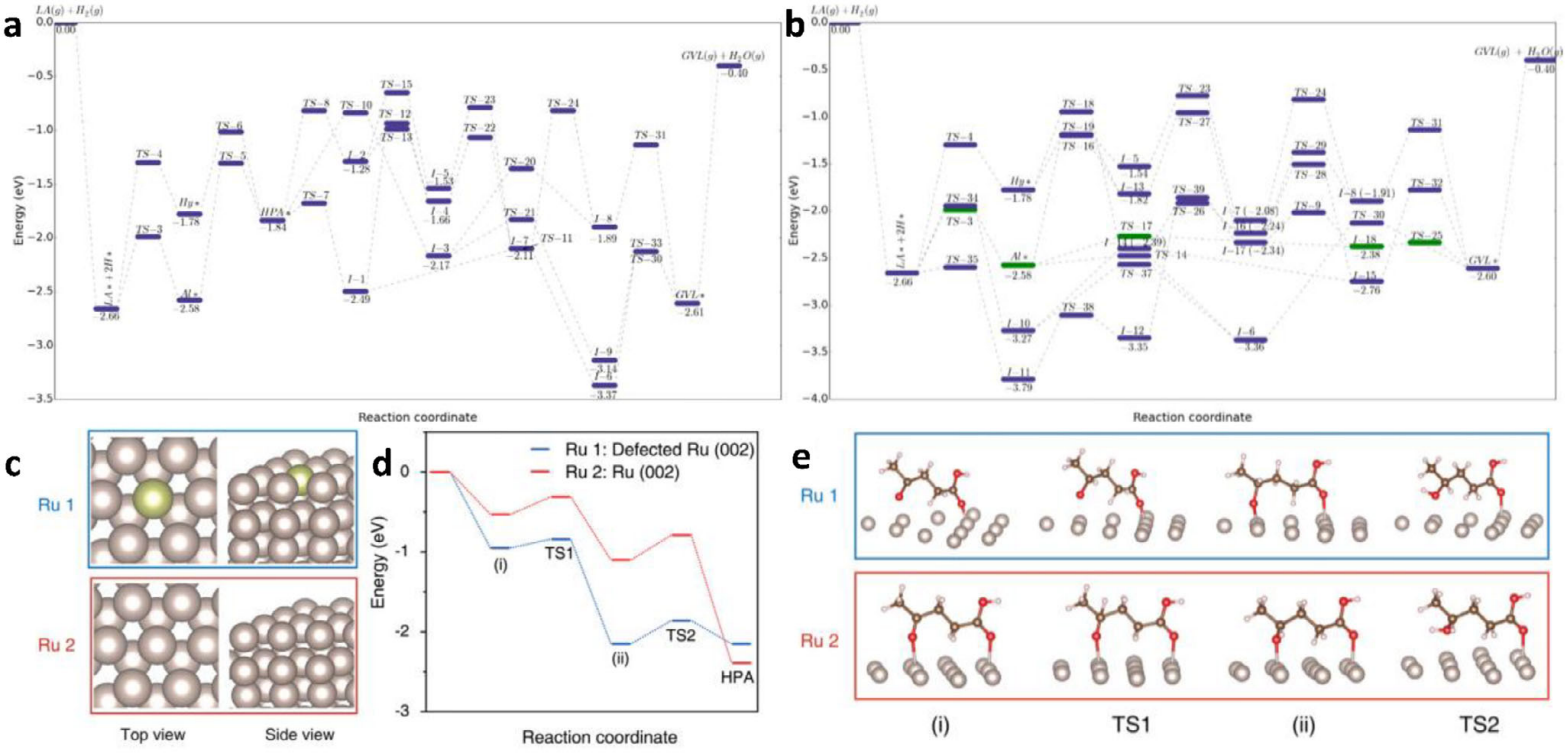
Zero‐point corrected energy diagram for the hydrodeoxygenation of LA over Ru(0001). (a) LA hydrogenation followed by dehydration pathways. (b) Direct catalytic conversion of LA to GVL. Stationary points in green are on the dominant reaction pathway. Reproduced with permission [34]. Copyright 2017, American Chemical Society. (c−e) On Ru1 defective clusters, the corresponding barriers for the addition of H^*^ on carbonyl C and carbonyl O in LA hydrogenation into 4‐HPA reduce to 0.09 and 0.29 eV, respectively, as compared to 0.22 and 0.31 eV on intact Ru2. Reproduced with permission [33]. Copyright 2024, Wiley.

Once 4‐HPA forms, catalyst acid sites drive intramolecular dehydration–esterification to yield GVL, releasing one water molecule. Reaction calorimetry by Delgado et al. measured the LA hydrogenation enthalpy over Ru/C as Δ*H* = −49.73 kJ mol^−1^, while the cyclization step was measured separately on the methyl 4‐hydroxypentanoate surrogate (Δ*H* = 9.39 kJ mol^−1^) because the 4‐HPA intermediate is unstable [36]. Comprehensive measurement results indicate that the overall reaction from LA to GVL is exothermic, whereas the lactonization step of this transformation is slightly endothermic. The role of the acidic sites must be confirmed through experiments to verify that they indeed influence the reaction rate.

For pathway 2 (dehydration–hydrogenation), LA first undergoes acid‐catalyzed intramolecular dehydration to *α*‐AL, followed by isomerization to *β*‐AL and subsequent hydrogenation to GVL. The initial dehydration step is kinetically demanding and is therefore favored at elevated temperatures, typically above 200°C, in the presence of acid sites. *α*‐AL can then isomerize to the thermodynamically more stable *β*‐AL. Theoretical calculations for related lactone systems indicate that this isomerization is thermodynamically favorable, with a reported Gibbs free‐energy change of approximately −32.22 kJ mol^−1^ at 25°C [20]. The C═C bond of *β*‐AL is subsequently hydrogenated at metal sites such as Ru to form GVL, and this hydrogenation step is strongly exothermic. These results indicate that pathway 2 combines a kinetically demanding initial dehydration with thermodynamically favorable downstream transformations. However, the thermodynamic driving force of the latter steps does not by itself determine pathway dominance or GVL selectivity. The GVL selectivity exceeding 99% reported in selected high‐temperature systems should instead be attributed to the combined effects of temperature, catalyst acidity, hydrogenation activity, residence time, and suppression of downstream GVL conversion.

Regarding further conversion of GVL, Rozenblit et al. provided a more refined analysis of the GVL ring‐opening network on Ru/C [37]. Under 200°C and 3.4–6.9 MPa H_2_, combined DFT and batch‐reactor experiments showed that GVL binds to Ru(0001) with a high adsorption energy of −141 kJ mol^−1^. Cleavage of the C1─O1 bond (the ring‐ether bond, distinct from the C1─O2 bond studied by Bababrik [11]) carries an energy barrier of only 7 kJ mol^−1^ with an exothermicity (−74 kJ mol^−1^), indicating that ring opening itself is not the RDS. The resulting acyl intermediate then competes along three parallel pathways: decarbonylation at an energy barrier of 96 kJ mol^−1^, hydrogenation at 89 kJ mol^−1^, and C─O hydrogenolysis with the highest barrier (149 kJ mol^−1^) [37]. Although decarbonylation is not the highest‐barrier route, it is most sensitive to CO poisoning. As CO surface coverage on Ru increases, the decarbonylation pathway is selectively suppressed and the hydrogenation pathway governs product distribution (Figure 5a) [37]. These findings complement Bababrik's C1−O2 cleavage route with an activation barrier of 25 kJ mol^−1^ [11], and together the two studies illuminate two distinct ring‐opening positions of GVL on Ru(0001), collectively mapping the complete picture of GVL deep conversion on the Ru surface. Both computational studies are limited to the idealized flat Ru(0001) surface. Barriers at low‐coordination B5‐type sites on real catalysts will deviate systematically, and direct experimental validation of this discrepancy is currently lacking.

**FIGURE 5:**
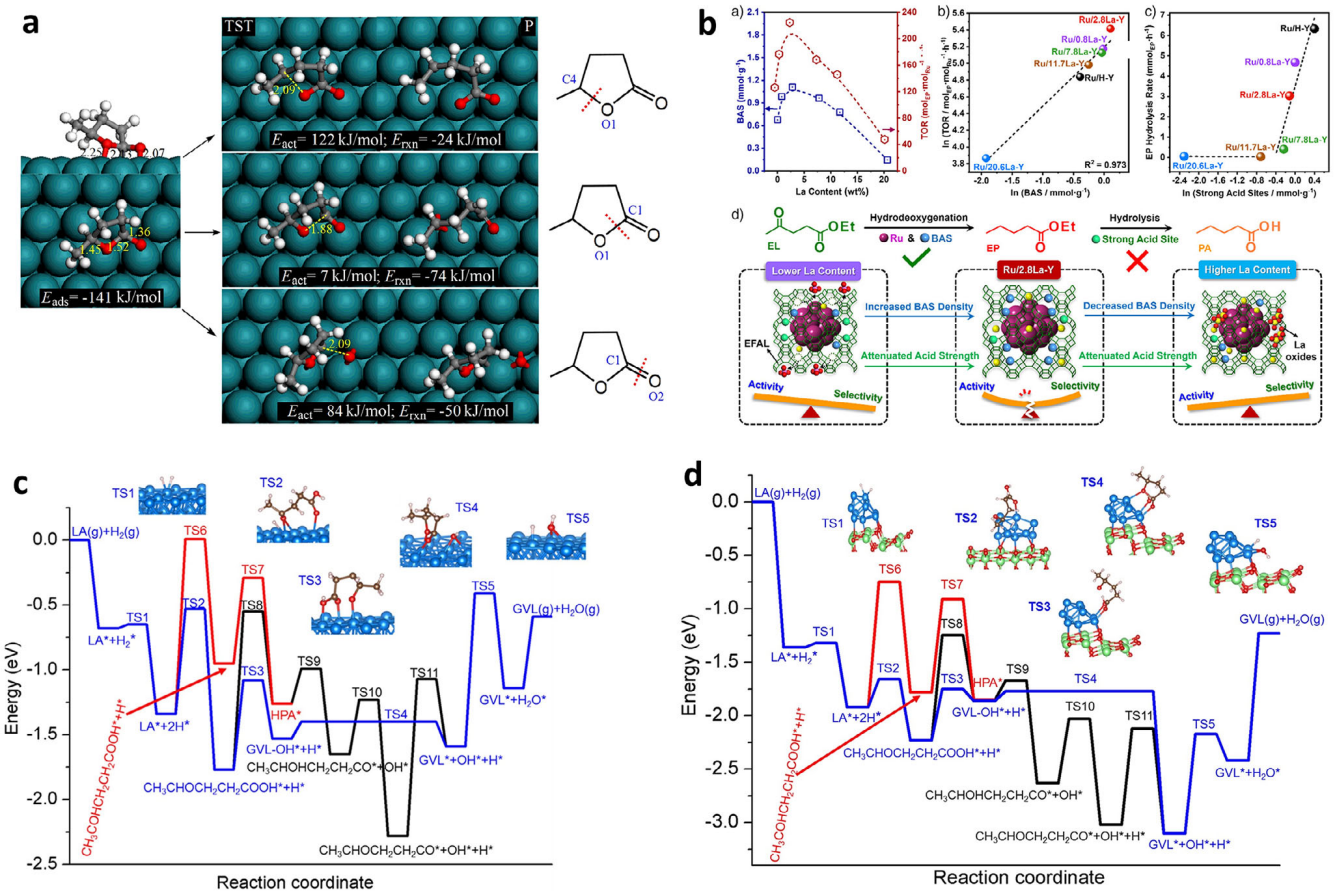
(a) DFT optimized adsorption geometry of GVL on Ru(0001) and its possible C─O breaking reaction paths. Reproduced with permission [37]. Copyright 2016, Elsevier. (b) Dual impact of Brønsted acid sites density on product distribution of EL hydrodeoxygenation. Ethyl pentanoate yield correlates positively with the density of Brønsted acid sites at *R* ^2^ = 0.973, yet elevated Brønsted acid sites simultaneously accelerates ethyl pentanoate hydrolysis to byproduct pentanoic acid. Reproduced with permission [41]. Copyright 2025, Wiley. (c,d) Comparison of hydrogenation and cyclization barriers at Ru/TiO_2_ interface. Reproduced with permission [23]. Copyright 2019, Elsevier.

### 2.2 Kinetic Features

The usage of the term “RDS” is inconsistent in literature. It is essential to distinguish between three concepts, that is, the highest energy barrier along a specific surface reaction step determined by DFT, the RDS identified via a kinetic model, and the reaction step that dictates the reaction rate within the constraints of the reactor system. As these concepts differ, they cannot be directly compared.

Abdelrahman et al. conducted a systematic kinetic investigation of pathway 1. Studies on Ru/C in the aqueous phase revealed that, below 150°C, the dehydration–lactonization of 4‑HPA constitutes the RDS of the overall reaction [38]. At temperatures exceeding 150°C, pore diffusion becomes the limiting factor. Crucially, this observation should be contextualized within the specific aqueous‑phase Ru/C system under study, extrapolating these findings to other catalysts or reactor configurations requires prior verification that gas‐liquid mass transfer, as well as external and internal diffusion effects, are negligible. In this reaction pathway, support acidity can significantly modulate the relative rates of hydrogenation and cyclization. Brønsted acid sites promote the dehydration–cyclization step through protonation of hydroxyl groups, whereas Lewis acid sites stabilize oxygenated intermediates or transition states via coordination. However, excessively high acid‑site density or acid strength is not always advantageous. In certain acidity‑tuned systems, a volcano‑shaped relationship has been observed between acid‑site density and GVL yield, that is, insufficient acidity renders the dehydration of 4‑HPA a kinetic bottleneck, while excessive or overly strong acidity may trigger GVL ring‑opening to valeric acid or further conversion to 2‑MTHF [39]. Abusuek et al. quantitatively examined this effect in HZSM‑5‑based systems and established a significant positive correlation between LAS density and the hydrogenation rate of GVL precursors, thereby confirming the promotional role of LAS in GVL formation [40]. Similarly, Lin et al. investigated the hydrodeoxygenation of EL to ethyl valerate (EV) and unveiled a dual regulatory effect of BAS, that is, increasing BAS density enhanced EV yield, while simultaneously accelerating the hydrolysis of EV into the by‐product valeric acid at a comparable linear rate (Figure 5b) [41]. This finding, in turn, substantiates the inherent selectivity penalty imposed by strong BAS toward the desired product.

Furthermore, supports can alter the RDS by modifying the metal‐support interface. Gao et al. demonstrated that Ru–O–Ti bonds at the Ru/TiO_2_ interface shift the RDS from the initial hydrogenation step on the Ru(002) facet to the cyclization step, thereby reducing the activation barrier from 0.81 to 0.48 eV (Figure 5c,d) [23]. While this result highlights the ability of support‑induced interfacial structures to modify the relative energy barriers of elementary steps, it does not imply that a change of support will necessarily alter the RDS. Rather, it underscores the critical need to explicitly report catalyst structures, acidity characterization parameters, assessments of mass‑transfer limitations, and the kinetic or computational methodologies employed, before making any comparative assignment of the RDS across different catalytic systems.

Compared with pathway 1, pathway 2 carries higher overall activation barriers and is kinetically unfavorable at low temperatures. In high‐temperature gas‐phase systems, however, its rate can surpass the 4‐HPA pathway to become dominant [42]. DFT calculations by Bababrik et al. further revealed that GVL ring‐opening via C(1)─O(2) bond cleavage on Ru(0001) carries an activation barrier of only 25 kJ mol^−1^ and is exothermic with Δ*H* = −31 kJ mol^−1^ [11], indicating that GVL possesses an intrinsic ring‐opening driving force at metallic Ru sites and is not unconditionally stable under high‐temperature or high‐H_2_‐pressure conditions. This finding provides an important kinetic basis for pathway control and operating‐condition selection.

Although temperature interacts with other factors such as reaction phase, solvent, and catalyst acidity, it remains a key determinant of the two competing pathways. In the low‑temperature liquid phase, the reaction generally favors the hydrogenation–lactonization route via the 4‑HPA intermediate. At high temperatures, particularly in the gas phase, the dehydration–hydrogenation pathway via the AL intermediate becomes preferred. This kinetic conclusion, which is specific to particular system, does not overturn the general trend of pathway with increasing temperature. Therefore, when designing catalysts for low‑temperature liquid‑phase operation, one should seek a balance between carbonyl hydrogenation and 4‑HPA lactonization. It cannot be assumed that higher acidity is invariably beneficial. At elevated temperatures, the accelerated enolization and dehydration steps enhance the contribution of pathway 2. However, excessively high temperatures may also promote GVL ring‑opening and subsequent hydrogenation reactions [31, 32, 35]. Reaction solvent also modulates pathway selection. Aqueous systems, where water molecules facilitate proton transfer, favor pathway 1, whereas solvent‐free or organic‐solvent systems better support the accumulation of pathway 2 intermediates. Consequently, catalyst design must adapt to the operational regime: for low‐temperature and liquid‐phase processes, design should prioritize balancing 4‐HPA formation with cyclization by enriching acid sites; for high‐temperature or gas‐phase processes, the primary design rule is to suppress over‐conversion of AL intermediate and prevent GVL ring‐opening [21].

Water molecules in the LA hydrogenation to GVL process are far from inert solvent but actively participate in the catalytic cycle. Using D_2_O isotope labelling in a Ru/TiO_2_ system, Tan et al. detected deuterium at the C5 position of GVL and confirmed that this deuterium was originated from O─D bond cleavage in D_2_O after excluding H/D exchange between GVL and D_2_O, proving that hydrogen in water molecules directly participates in LA carbonyl hydrogenation [43]. Michel et al. revealed the molecular mechanism theoretically, showing through DFT calculations that water molecules adsorbed on the Ru surface lower the carbonyl hydrogenation barrier through hydrogen‐bond by approximately 0.41 eV [44]. The fundamental reason is that the strong oxophilicity of Ru stabilizes adsorbed water as surface hydroxyl species, which provide an additional low‐energy channel for C═O hydrogenation via proton transfer. The less oxophilic Pt catalyst does not display this water‐promotion effect. This water‐assisted proton transfer mechanism explains why the Ru‐based system achieves higher hydrogenation rate in aqueous media than in organic solvents under comparable reaction conditions. Microkinetic modeling by Mamun et al., based on Ru(0001), quantitatively confirmed that at 50°C the aqueous‐phase reaction rate is approximately 35‐fold higher than in 1,4‐dioxane and roughly four orders of magnitude higher than in the gas phase. This promotion effect weakens with increasing temperature [35]. Using an aqueous medium under mild low‐temperature conditions is therefore one of the most direct and cost‐effective engineering options for enhancing Ru‐based catalyst activity [43, 44].

Efficient progression along both pathways relies on precise cooperation between metallic hydrogenation sites and acidic cyclization sites. Ru^0^ provides a high density of reactive H^*^, while BAS/LAS drive 4‐HPA dehydration–cyclization or initial enolization–dehydration of *α*/*β*‐AL. Imbalanced metal–acid synergy directly shifts the product distribution. Excessive hydrogenation capacity coupled with insufficient acid sites leads to 4‐HPA accumulation or C─C bond cleavage to VA. Excessive acidity with insufficient Ru hydrogenation sites drives LA/GVL ring‐opening to VA and its derivatives under strong acid conditions. When high H_2_ pressure coexists with strong Ru activity, GVL can be further hydrogenated to 2‐MTHF [39, 45]. Catalyst design should optimize both hydrogenation activity and acidity. Equally important is the proximity between metal and acid sites, because efficient transfer of intermediates such as 4‐HPA between them directly affects the net reaction rate.

For selective hydrogenation of levulinate ester substrates, the reaction mechanism differs markedly from that of LA in two respects, active site identity and RDS attribution. In situ CO diffuse reflectance infrared spectra (in situ CO‐DRIFT‐IR) by Dao et al. clearly established that on the Ru/LaQS catalyst, Ru^3+^ rather than conventionally assumed metallic Ru^0^ is the key activation site for EL conversion via Lewis acidity, despite Ru^0^ exhibiting the stronger adsorption for EL than Ru^3+^ (−2.123 eV vs. −1.419 eV). The Lewis‐acid character of Ru^3+^ drives C─O bond cleavage rather than substrate adsorption [46]. The RDS for EL conversion is C─O single‐bond activation rather than C═O carbonyl hydrogenation, as evidenced by C─O bond elongation from 1.441 Å to 1.489 Å in the 4‐hydroxyvalerate (4‐HPE) intermediate upon adsorption. Under this mechanism, the reaction follows EL to 4‐HPE to GVL, analogous to LA to 4‐HPA to GVL, but with a more prominent contribution from LAS‐mediated hydrogen‐transfer pathway because EL lacks a free carboxyl group available for direct protonation. Cooperative action between Ru^0^ and Ru^3+^ dual‐active sites is key to achieving high EL conversion and GVL selectivity, which is the central mechanistic finding in the work of Dao et al. [46].

The macroscopic kinetics of LA hydrogenation to GVL broadly conform to the Langmuir–Hinshelwood (L–H) model, and experimental kinetic data show typical non‐competitive adsorption between LA and H_2_, with the two species occupying distinct types of active sites and not competing directly [38]. The LA concentration dependence of the reaction rate approximates first‐order kinetics. The reported reaction order of H_2_ varies significantly, ranging from approximately 0.07 to 0.70 [34, 35, 38]. These values were obtained under different H_2_ pressure ranges, catalysts, solvents, conversions, and kinetic models. Therefore, they should not be interpreted as a single trend. In some kinetic and theoretical studies, as H_2_ pressure increases or surface H coverage rises, the contribution of the hydrogenation step to the overall reaction rate decreases, thereby increasing the contribution of the dehydration‐cyclization step to the overall reaction rate. Determining whether such a change in the RDS occurs requires assessment that is specific to both the catalyst and the H_2_ pressure range being investigated. Using DFT calculations and in situ Fourier transform infrared spectroscopy (in situ FTIR), Shao et al. showed that LA undergoes preferential hydrogenation at the *γ*‐C position to form an alkoxide intermediate, which cyclizes directly to GVL [25]. This result provides molecular‐level evidence for preferential *γ*‐C hydrogenation under the investigated conditions, but it does not independently establish a non‐competitive Langmuir–Hinshelwood adsorption model. Protsenko et al. studied LA hydrogenation over 5% Ru/MN100 [47]. Formal kinetic modelling at 80°C–110°C and 1–5 MPa selected a dual‐site, non‐competitive L–H model as the best fit. The H_2_ reaction order is 0.7, and the apparent activation energy is 48–63 kJ mol^‒1^. Molleti et al. examined LA hydrogenation over 1 wt% Ru on manganese oxide octahedral molecular sieves (OMS‐2) at 80°C–110°C and 10–40 atm H_2_ [48]. A non‐competitive Langmuir–Hinshelwood–Hougen–Watson rate expression fits the data best. The apparent activation energy is about 49 kJ mol^‒1^. Delgado et al. used a Bayesian framework to rank seven candidate rate laws for the co‐hydrogenation of LA and n‐butyl levulinate over 5% Ru/C at 100°C–141°C and 0.95–2.2 MPa [49]. The two most probable models were both non‐competitive, with LA, ester, and H_2_ on separate Ru sites. Data obtained from these experiments should not be taken as evidence for the existence of different RDS. Similarly, the fitted H_2_ reaction order alone cannot be used to determine the RDS, because it varies with the H_2_ pressure range, H_2_ surface coverage, catalyst structure, solvent, mass transfer effects, conversion, and the chosen rate expression. Therefore, a more accurate H_2_ reaction order should be derived from initial rate measurements conducted within a specified H_2_ pressure range, after mass transfer limitations have been ruled out.

Existing studies indicate that the sensitivity of the reaction rate to H_2_ pressure tends to decrease with increasing H_2_ coverage. Low and high coverages are defined relative to a given catalyst and solvent system, and are not absolute across different systems (Table 1). For similar catalyst structures, the fitted H_2_ reaction order can be directly compared only when mass transfer limitations are absent. The exponents in rate equations are generally model‑dependent. Therefore, the H_2_ order derived from the L‑H model or from other specific kinetic equations still depends on the chosen model and the reaction system under consideration.

**TABLE 1:** The general relationship between H_2_ pressure and catalytic performance.

H_2_ pressure range	Relationship between reaction rate and H_2_ pressure	Kinetic explanation	Solution
Low pressure	Shows a strong positive correlation; the reaction is nearly first‐order	Low H_2_ availability or low reaction rates in H_2_‐related reaction steps	Fit the local apparent reaction order within the defined low‐pressure range
Medium pressure	Shows a fractional‐order reaction, which weakens as pressure increases	As H_2_ coverage increases, sensitivity to H_2_ gradually decreases	Report local sensitivity coefficients or fit a saturated model
High pressure	Shows a weak correlation; the reaction is nearly zero‐order	The weak dependence of the reaction rate on hydrogen pressure may reflect an increase in H_2_ coverage or an increase in the relative contribution of other steps.	Report local sensitivity coefficients at saturation or when the reaction order approaches zero, rather than extrapolating the low‐pressure reaction order

### 2.3 Hydrogen Sources for Hydrogenation

Hydrogen source directly determines the greenness, cost structure, and process safety of the catalytic system. Two principal strategies currently exist, that is, externally supplied molecular hydrogen and CTH.

External H_2_ is employed most extensively. Its advantages include a stable hydrogen source, well‐defined reaction conditions, and avoidance of catalyst poisoning by impurities. However, strict equipment requirements for high‐pressure H_2_ storage and transport create significant safety and cost burdens during industrial scale‐up, motivating continued exploration of alternative hydrogen sources. FA, co‐produced in near‐equimolar quantities with LA during acid hydrolysis, offers inherent process‐integration advantages and is one of the most widely studied CTH hydrogen donors [50, 51]. In FA‐donor CTH systems, FA adsorbs on Ru surfaces more strongly than LA, preferentially occupying active sites and suppressing effective LA activation [52]. FA decomposition generates H^*^ via the dehydrogenation route in which HCOOH decomposes to H_2_ and CO_2_, or produces CO via the dehydration route, poisoning Ru active sites [53]. GVL productivity depends strongly on selectivity toward FA dehydrogenation rather than dehydration, because the latter generates CO that can poison Ru sites, where Ru holds an intrinsic advantage over Pd [54]. Therefore, the corrosiveness and CO poisoning risk of FA‐donor CTH systems are the main engineering constraints [55].

The MPV reduction route using alcohols as hydrogen donors is considered a more practically promising CTH strategy owing to its low cost, safety, and non‐corrosive character [56]. Primary or secondary alcohols dehydrogenate to the corresponding aldehydes or ketones, transferring hydrogen to reduce the substrate. Alcohol‐donor CTH generally proceeds in three steps: esterification of LA with the alcohol to form a levulinate ester, reduction of the C═O bond in the ester by the active hydrogen of the alcohol to give 4‐hydroxy levulinate, and departure of the alcohol molecule accompanied by cyclization of 4‐hydroxy levulinate to GVL (Figure 6) [57, 58, 59, 60]. Secondary alcohols exhibit superior hydrogen‐transfer capability relative to primary alcohols due to lower redox potential [61, 62]. As alcohol carbon chain length increases, declining solvent polarity and increasing steric hindrance both reduce LA conversion and GVL yield [63]. Chia and Dumesic first reported liquid‐phase conversion of LA and its esters to GVL via alcohol hydrogen transfer over metal oxide catalysts [64]. Subsequent studies by Yu et al. and Wang et al. demonstrated that the synergistic cooperation between Brønsted/Lewis acid sites and basic sites on the catalyst surface drives both H_2_ generation and hydrogen transfer in the CTH process [65, 66]. Under continuous gas‐flow conditions, Tabanelli et al. identified a third reaction pathway for alkyl levulinates, that is, on ZrO_2_, alkyl levulinates can first undergo intramolecular alcoholysis–dealkylation to *α*/*β*‐AL intermediates, which are then reduced to GVL by the alcohol hydrogen donor. This AL intermediate route operates more efficiently under continuous‐flow conditions, and basic‐site‐catalyzed cyclization of the substrate is indispensable, with Lewis acid and basic sites acting synergistically [67]. The RDS in alcohol‐source CTH differs from that in direct H_2_ hydrogenation, a systematic review of CTH systems by González et al. noted that while some literature identified C─H bond formation at the *γ*‐C position as rate‐controlling, their analysis of reported data suggests that esterification or cyclization is the overall determining step, as maximum conversion is reached significantly earlier than maximum GVL selectivity. The cyclization is generally considered to occur readily, especially given that 4‐HPA has lower steric hindrance than its ester counterparts [68]. This interpretation is consistent with the preferential *γ*‐C hydrogenation reported by Shao et al. [25], but preferential reaction at the *γ*‐C position does not by itself establish that this step controls the overall rate. Depending on the substrate, catalyst, and analytical method, the inferred rate‐controlling transformation may instead involve esterification, hydrogen transfer, cyclization, 4‐HPA dehydration, or Ru^3+^‐mediated C═O activation [23, 38, 46, 68]. Accordingly, RDS assignments in alcohol‐mediated CTH should be restricted to the catalyst and operating regime in which they were derived. Collectively, these studies show that rate‐control assignments are specific to the substrate, catalyst structure, operating regime, and method used.

**FIGURE 6:**
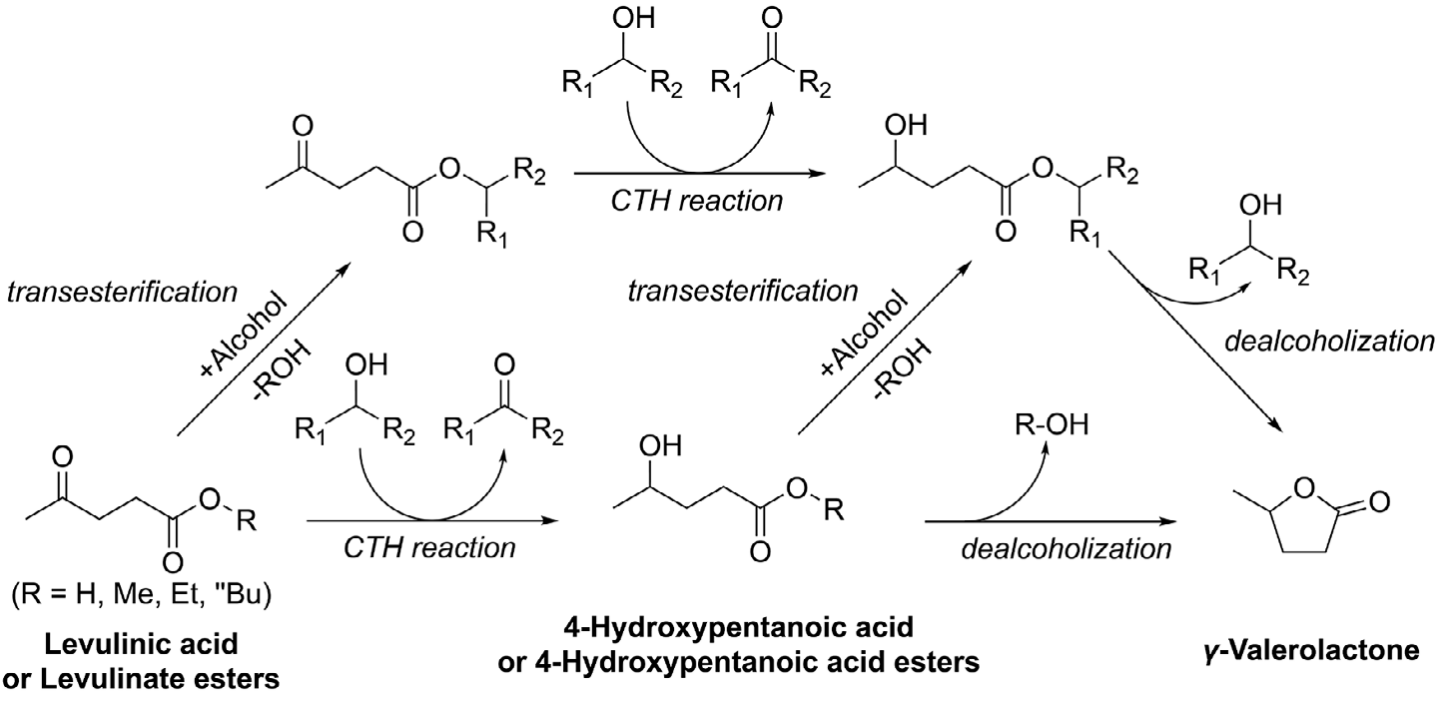
CTH of LA and its esters using alcohol as a H‐donor. Reproduced with permission [57]. Copyright 2017, Elsevier.

### 2.4 Phase Selection of Catalytic Systems

In homogeneous catalytic systems, strong coordination interactions between metal complexes and substrates often deliver extremely high GVL yield. Jurado‐Vázquez et al. reported a system based on a homogeneous copper(I) complex with H_2_, achieving 99% GVL yield after 24 h at 140°C [69]. Similarly, a homogeneous Ni catalyst with FA as hydrogen source reached 99% GVL yield after 4 h at 120°C [70]. However, homogeneous catalysts suffer from inherent drawbacks, including expensive ligands, difficult catalyst–product separation, severe equipment corrosion, and inability to operate continuously, all of which render them economically unattractive at modest production scales.

Heterogeneous catalysts allow rapid catalyst–product separation by simple filtration or centrifugation, significantly reduce separation costs, and facilitate catalyst recycling and regeneration, making them better suited for industrial‐scale application and the current mainstream research direction. Bourne et al. exploited a biphasic supercritical CO_2_–water system with Ru/SiO_2_ for aqueous‐phase LA hydrogenation, achieving 99% GVL yield, and near 100% purity GVL is recovered upon CO_2_‐phase depressurization [71]. However, this system requires approximately 10 MPa operating pressure, imposing demanding equipment requirements. Supported Ru catalysts operating under mild conditions, combining high activity with regenerability, have become the dominant system for heterogeneous catalytic GVL production.

## 3 Multiscale Regulation Strategies for Ru‐Based Catalysts

The conversion of LA and levulinates to GVL involves steps such as H_2_ activation, C═O activation, active hydrogen transfer, and lactonization. The relative importance of these steps varies depending on the substrate, hydrogen source, solvent, and temperature [34, 35, 38, 46, 68]. Consequently, catalyst performance cannot be explained by a single structural characteristic alone. The electron density of Ru affects H_2_ and C═O activation. The acid‐base sites affect intermediate transformation. The support impacts Ru dispersion, interfacial structure, and reactant adsorption and diffusion. The reaction solvent influences surface reactant coverage and intermediate stability. Complex interplay exists between these effects. For instance, N‐ or P‐containing supports can simultaneously modify Ru electron density, dispersion, surface acid‐base properties, and hydrophilicity [72, 73, 74], while confinement effects may alter local reactant concentrations and diffusion behaviours [75]. In light of this, this section explores five interrelated regulation aspects: electronic structure of Ru and acid sites, support structure and morphology, particle size and dispersion, reaction conditions and solvent microenvironment, and resistance to deactivation (Table 2). It should be noted that due to variations in substrates, hydrogen sources, conversion ranges, and normalized activity definitions, a direct comparison of catalytic performances across different studies is not feasible. Therefore, this section compares representative results only under the specific reaction conditions reported in each study.

**TABLE 2:** Reaction limitations and optimization strategies for Ru‐catalyzed conversion of LA and levulinates to GVL.

Reaction limitation	Potential limiting steps	Measurable parameters	Optimization strategies
Intrinsic rate changes reproducibly with H_2_ pressure and shows a H_2_/D_2_ isotope effect after mass‑transfer exclusion	H_2_ activation or hydrogen‐addition contributes to rate control	H_2_ order, kinetic isotope effect, Ru coordination or ensemble density, Ru structure under working‐state	Electronic structure, particle size and dispersion, metal‐support interface
4‐HPA accumulates and its conversion is sensitive to acid‐site perturbation	Cyclization or lactonization contributes to rate or selectivity control	4‐HPA transient concentration, independent 4‐HPA conversion, acid amount and strength, metal‐acid proximity	Acid‐site regulation, support or interface modification, solvent microenvironment
Rate or selectivity changes with agitation, particle size, and flow rate	External or internal transport limitation	Agitation test, particle‐size test, Mears criterion, Weisz–Prater criterion	Support morphology, pore architecture, reactor or operating conditions
LA conversion decreases with increasing FA concentration or with increasing CO coverage	FA/LA competition or CO poisoning	FA decomposition route, adsorbed CO, donor/substrate ratio, LA‐conversion response to FA concentration	Hydrogen‐source and condition regulation, Ru/support electronic regulation
Activity loss correlates with dissolved Ru, particle growth, support transformation, or pore blockage	Leaching, sintering, support instability, or fouling	Ru loss, particle size, support phase, carbon balance	Deactivation control, support stabilization, particle‐size regulation
Real‐feed productivity falls relative to model feed	Impurity poisoning, fouling, or feed‐induced structural instability	Impurity‐response tests, productivity loss, Ru loss, pressure drop, regeneration efficiency	Deactivation control, support architecture, process‐condition adjustment

### 3.1 Electronic Structure and Acid Site Regulation

The analysis in section 2 indicates that modulating the electronic state of Ru and the nature of acid sites can effectively overcome limitations associated with the two reaction mechanisms. Ru sites govern H_2_ activation and the hydrogenation of C═O bonds, whereas acid sites facilitate 4‐HPA lactonization, AL formation, or LA activation, depending on the specific substrate and hydrogen source [34, 38]. Consequently, the strategy of regulating the electronic structures and acid sites should be prioritized only after mass‐transfer effects have been ruled out and the evidences such as kinetic data or the detection of intermediates point to the aforementioned limitations. Relevant recognizable parameters include the dependence on H_2_ pressure, the transient concentrations of 4‐HPA or AL, the type and strength of acid sites, and the spatial proximity between Ru sites and acid sites. Besides, since the introduction of heteroatoms can simultaneously alter the factors such as the electronic state and dispersion of Ru, support structure, hydrophilicity, and support acidity/basicity, a shift in binding energy alone is insufficient to prove that enhanced activity stems from electronic effects. In several carbon‐supported systems modified with N, P, or B, the introduction of heteroatoms is linked to electron redistribution at the Ru sites. Notably, pyridinic‐N induces electron transfer from the support to Ru, thereby generating electron‐rich Ru^0^ species [72, 73]. DFT calculations confirm that electron‐enriched Ru centers substantially lower the H_2_ heterolytic dissociation and C═O activation barriers, enhancing carbonyl adsorption‐polarization and intrinsic hydrogenation activity. Meng et al. systematically investigated the metal‐support interaction in N‐doped carbon‐supported Ru catalysts for LA hydrogenation to GVL, demonstrating that heteroatom‐induced Ru electron enrichment enhances hydrogenation activity [72].

Cooperative action between BAS and LAS is critical for dehydration‐lactonization of the 4‐HPA intermediate. Acid strength regulation involves an inherent trade‐off, since excessively strong acidity not only promotes targeted GVL cyclization but also drives GVL ring‐opening to valeric acid or over‐hydrogenation to 2‐MTHF, reducing target‐product selectivity [39]. Introducing alkali metal promoters such as K and Na, or organic bases such as triethylamine, adjusts the acid‐base balance to suppress deep GVL conversion and finely tunes product distribution. For levulinate ester substrates, LAS‐mediated hydrogen transfer contributes more prominently, while the alcohol‐donor CTH process depends heavily on acid‐base pair cooperation at the catalyst surface [64, 65, 66].

Bounoukta et al. grafted aminosilane onto carbon nanofiber (CNF‐APTES) surfaces via an organometallic approach, constructing N−Ru cooperative active sites. Pyridinic N transfers electrons to neighboring Ru sites, creating electron‐rich Ru^0^ that promotes π‐backbonding to the LA carbonyl. Ru/CNF‐APTES achieved a turnover frequency (TOF) of 9887 h^‒1^ at 100°C and 1.0 MPa H_2_ [76]. Ru/CNF‐ILs with imidazolium ionic liquids, showed only 36% dispersion yet exhibited a higher TOF of 17 222 h^‒1^, because N sites participate directly in surface hydrogen activation via an ion‐pair effect, independent of metal dispersion. Cai et al. prepared Ru/*g*‐C_3_N_4_ catalysts in which the C–N═C framework of *g*‐C_3_N_4_ donates electrons to Ru through an electron‐donation effect, generating electron‐rich Ru^0^ [77]. CO‐DRIFTS confirms that increased electron density favors π‐backbonding activation of the substrate C═O. Basic N sites simultaneously drive FA dehydrogenation in which HCOOH decomposes to H_2_ and CO_2_, effectively preventing CO poisoning. In an isopropanol‐donor (IPA‐donor) CTH system, 100% LA conversion and 99.8% GVL yield were achieved under 100°C.

Han et al. prepared Ru_1_@WO_x_/CN by anchoring single‐atom Ru to uniform WO_x_ sub‐clusters loaded onto a N‐doped carbon surface via a missing‐site polyoxometalate (N‐POM) strategy [78]. In this system, the Ru center exists in a highly dispersed, tetravalent single‐atom form. EXAFS shows only a Ru–O peak at 1.53 Å, with no Ru–Ru peak at 2.33 Å, confirming the absence of metal clusters or nanoparticle structures. This catalyst exhibits excellent synergy, with the electronic structure of the Ru active center being jointly modulated by the WO_x_ clusters and the CN substrate, whilst the acidified WO_x_ is responsible for promoting the conversion of the 4‐HPA intermediate during reaction. Under solvent‐free conditions at 100°C and 2.0 MPa H_2_, the system achieved a LA conversion over 99% and 100% GVL selectivity, with a TOF value of 1060 h^‒1^, significantly outperforming conventional Ru/C and single‐atom Ru_1_/CN catalysts. Ruiz‐Bernal et al. examined the effect of thermal treatment temperature on activated carbon‐supported Ru catalysts for LA hydrogenation [79]. Treatment at 900°C removes the majority of oxygen‐containing functional groups. The resulting hydrophobic surface enables complete Ru^0^ reduction. After H_2_ dissociation at Ru^0^ sites, hydrogen spillover generates Brønsted acid sites in situ at residual RuO_x_ sites, directly driving 4‐HPA dehydration‐lactonization. This hydrogenation‐driven acid‐site self‐generation mechanism improved LA conversion and GVL selectivity in aqueous phase at 70°C, with LA conversion ranging from 11% to 84% depending on the carbon support surface chemistry.

Abusuek et al. prepared Ru catalysts on HZSM‐5 supports with Si/Al ratios of 40, 80, and 400, proposing a dual‐site cooperative mechanism between RuO_2_ and Lewis acid sites [40]. RuO_2_ itself functions as an efficient active phase, with its LAS adsorbing C═O while in situ ‐generated trace Ru^0^ handles H_2_ dissociation, operating non‐competitively. Quantitative linear relationships were established between LAS intensity, reaction rate, and surface RuO_2_ content. The optimal Ru/HZSM(40)‐5 achieved 98% LA conversion and 100% GVL selectivity at 100°C and 1 MPa H_2_, with an apparent activation energy of 46 kJ mol^‒1^. Yi et al. systematically compared product distributions over Ni and Ru on HZSM‐5 support. Ni/HZSM‐5 produced GVL as the major product in 93.1% yield [80]. Ru/HZSM‐5, by contrast, owing to its strong ability to ring‑open and hydrodeoxygenate GVL, mainly afforded valeric acid and valerate esters (85.7% combined) with only 1.2% GVL. This contrast demonstrates that metal identity strongly dictates product selectivity under equivalent catalyst‑support and reaction conditions [80]. Xi et al. used 2‐pentanone as a LA model compound to examine how Si/Al ratios of 21–360 tune acid sites on Ru/HZSM‐5 [81]. LAS activates C═O to promote hydrogenation. BAS drives dehydration of 2‐pentanol. The two roles are non‐competing and cooperative. The optimal catalyst with Si/Al ratio of 21 achieved 91.8% conversion and 77.7% *n*‐pentane selectivity at 190°C, 5.0 MPa H_2_, and 6 h.

Koley et al. constructed a Ru/HPW bifunctional system using Keggin‐type phosphotungstic acid (HPW) as support [82]. The superstrong Brønsted acid sites of HPW lower the activation energy for dehydration‐cyclization by protonating the 4‐HPA hydroxyl group, forming a proton‐assisted tandem mechanism with Ru‐catalyzed C═O hydrogenation. X‐ray photoelectron spectroscopy (XPS) confirms Ru^0^ as the dominant phase. Ru–O–W bonds provide effective active‐site dispersion. GVL yield exceeded 90% under optimal conditions. Al‐khawlani et al. prepared Pt‐Ru bimetallic/Y zeolite catalysts using ethylene glycol reduction, with support acidity tuned by alkali‐thermal treatment [83]. The treatment dissolves Si in framework, lowers the Si/Al ratio, and generates extra‐framework Al as strong Lewis acid sites that accelerate 4‐HPA dehydration‐cyclization. XPS confirms that alkali‐thermal treatment simultaneously promotes Pt^2^^+^ reduction to Pt^0^, enhancing C═O hydrogenation activity. Pt–Ru synergy also preserved Y framework integrity during treatment. The optimal catalyst achieved complete LA conversion and 98% GVL yield at 160°C and 2 MPa H_2_, with no significant activity loss over three cycles. Lu et al. prepared Ru catalysts on Al_2_O_3_–ZrO_2_ mixed oxide supports via co‐precipitation [84]. The L/B ratio peaked at Al/Zr = 10:1, while the absolute Lewis acid site density decreased from 0.251 mmol g^‒1^ for pure Al_2_O_3_ to 0.029 mmol g^‒1^ for pure ZrO_2_. Al–O–Zr interfacial bonds strengthened metal–support interaction. The optimal Al/Zr of 10:1 composition delivered an L/B ratio of 46.3 and a high density of strong acid sites of 1.656 mmol g^‒1^. XPS confirms metallic Ru^0^ as the dominant species, while X‐ray diffraction (XRD) and transmission electron microscopy (TEM) indicate high Ru dispersion. Both LA conversion and GVL selectivity reached 100% at 120°C, 1.0 MPa H_2_, and 4 h, confirming that Lewis acid sites govern the 4‐HPA dehydration‐cyclization step. Kumaravel et al. prepared Ru catalysts supported on Sn‐modified SBA‐15 for the vapor‐phase hydrogenation of LA using molecular hydrogen as the reductant, achieving 99% conversion and 98% selectivity to GVL at 250°C [85]. Characterization by temperature‐programmed desorption of NH_3_ (NH_3_‐TPD) confirmed that the incorporation of Sn and Ru significantly enhanced the total acidity of the 2D hexagonal ordered mesoporous support. Conversely, the application of Sn‐modified SBA‐15 for CTH using IPA as the hydrogen donor was reported by Xu et al. [86]. Their study utilized Fourier transform infrared spectra of adsorbed pyridine (Py‐IR) to demonstrate that highly dispersed Sn species provided synergistic Lewis and Brønsted acid sites essential for the tandem steps of hydrogen transfer and dehydration or esterification. While the Sn‐modified catalyst delivered high GVL yield in the CTH system, the unmodified SBA‐15 support exhibited no detectable activity, as it lacks the necessary acid‐base sites to drive the hydrogen transfer process. Esposito et al. co‐deposited Nb_2_O_5_ and Ru onto SiO_2_ by sequential impregnation to prepare a Ru–Nb/SiO_2_ bifunctional catalyst [87]. The strong Lewis acid sites of Nb^5+^ coordinate‐activate the LA C═O bond. Nb−O−Ru interfacial bonds shift the Ru *d*‐band center, together forming a non‐competitive dual‐site cooperative mechanism. The optimal 4Ru‐2.5Nb/SiO_2_ achieved GVL selectivity >98% at 70°C and 2.0 MPa H_2_, with a TOF of 1.24 × 10^‒2^ s^‒1^, a 370‐fold improvement over Ru/C.

Grigorev et al. used hypercrosslinked polystyrene (HPS) as support for Ru–Co catalysts [88]. Co addition does not modulate Ru oxidation state through electron transfer. Instead, it redistributes Ru within the HPS matrix and increases mesoporosity. The inherent hydrophobicity of HPS preferentially enriches the LA substrate and promotes GVL desorption, enhancing mass transfer. Complete LA conversion with near 100% GVL yield was achieved in aqueous phase at 120°C, 2.0 MPa H_2_, and 60 min. Wang et al. prepared Ru catalysts on lignin pyrolysis‐derived hierarchical N‐doped carbon (LDMCN) [89]. Multiple N species cooperatively transfer electrons to Ru, forming electron‐rich Ru^0^. In a CTH system with IPA as solvent and hydrogen donor, Ru/LDMCN‐20% achieved complete LA conversion and 99.3% GVL yield, with GVL yield declining from 99.3% to 80.0% after five cycles. Zhu et al. and Korampattu et al. independently used furfural pyrolysis‐derived N‐C and chitosan‐derived N‐C as supports, respectively, both confirming that the electron‐donating effect of pyridinic N forms electron‐rich Ru^0^ and achieves efficient LA hydrogenation with good recyclability under mild conditions [90, 91]. Wang et al. further introduced N, P co‐doping to prepare ZIF‐8‐derived macroporous core‐shell Ru_2_P–Ru^0^@PN‐C. P doping shifts the *d*‐band center upward, enhancing H_2_ dissociation and C═O activation [74]. The TOF reached 20 339 h^‒1^ at 30°C and 1.0 MPa H_2_, with >99.9% GVL selectivity, maintaining >93% conversion over four cycles with structure and particle size preserved (Figure 7a). Petcuta et al. examined the effect of BAS density on LA hydrogenation using Beta zeolite supports with Si/Al ratios of 12.5–150, additionally probing the influence of synthesis method on Ru dispersion. The optimal 3Ru/BEA150 achieved 96.5% conversion and 97.8% GVL selectivity at 130°C, 1 MPa H_2_, and 24 h, using 1,4‐dioxane as solvent [92]. Nguyen et al. prepared Ru@FC catalysts using phosphoric acid‐activated biochar (FC) as support [93]. P‐ and O‐containing functional groups provide BAS that cooperates with Ru hydrogenation function. GVL yield reached 98.6% in an IPA‐donor CTH system, with stable activity over five cycles. Yang et al. synthesized a core‐shell Ru/MgSiO_3_@MFI catalyst by in situ coating MgSiO_3_ onto a MFI zeolite framework [94]. Basic Mg–O sites on MgSiO_3_ reduced the apparent activation energy for 4‐HPA cyclization from 57 to 18 kJ mol^‒1^. EL was completely converted and GVL selectivity reached 98% at 80°C and 0.5 MPa H_2_. This shows that basic sites can replace Brønsted acid sites to drive 4‐HPA dehydration‐cyclization.

**FIGURE 7:**
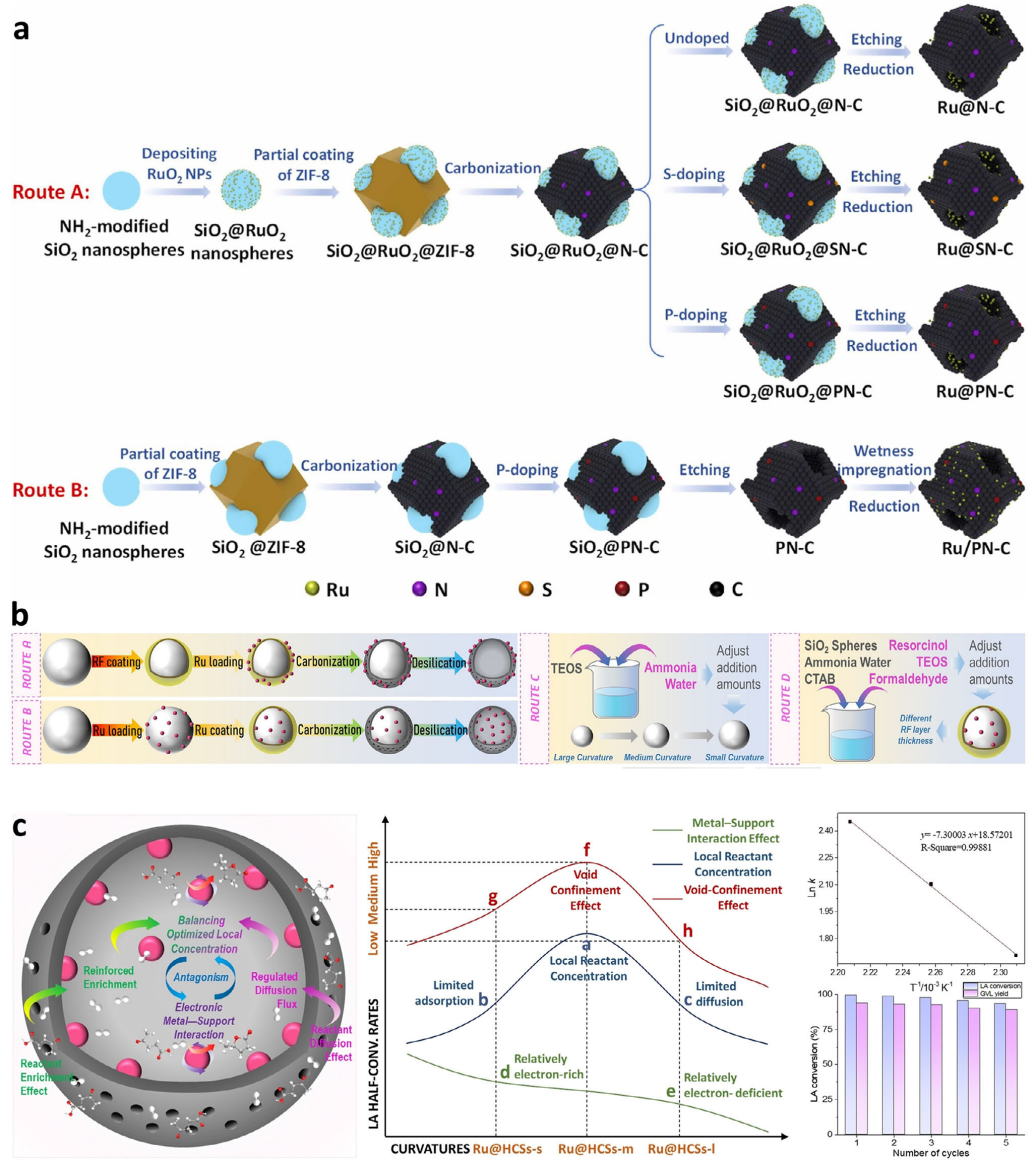
(a) Synthetic routes for macroporous core‐shell Ru@N‐C, Ru@SN‐C, and Ru@PN‐C catalysts (Route A) and externally supported Ru/PN‐C catalyst (Route B). Reproduced with permission [74]. Copyright 2026, Elsevier. (b) Synthetic routes of Ru‐loaded HCSs nanoreactor analogues. (c) Potential forming mechanism of the void‐confinement effect and its corresponding illustration, correlation between temperature and apparent rate constant of LA hydrogenation over Ru@HCSs‐m, and cycling test of LA hydrogenation over Ru@HCSs‐m. Reproduced with permission [75]. Copyright 2021, Wiley.

Up to now, N‐doping strategy still faces several challenges in practical application. First, the content and spatial distribution of pyridinic‐N are difficult to precisely control at the synthesis stage. XPS provides only surface ratios of N species and cannot resolve their spatial distribution around Ru nanoparticles. Second, long‐term chemical stability for N‐C supports under hydrothermal acidic conditions remains to be improved. Some reports show reduced N content after recycling, but quantitative analysis of the impact on catalytic activity is unsystematic. Third, the relationship between N‐doping electron‐donation effect and support acid sites varies by system, and a universal structure–activity model remains elusive. These limitations indicate that N‐doping regulation is still in a phenomenon‐driven stage, far from the rational design‐guided precision control. Overcoming this bottleneck requires operando XPS and related in situ techniques to track the dynamic evolution of N species under reaction conditions. While screened data can support the generation of exploratory hypotheses, constructing predictive models requires descriptive indicators based on consistent measurement standards, which are rarely reported in existing literature. Furthermore, the chemical stability of N‐C supports in hydrothermal acidic media should be adopted as an independent evaluation metric, rather than being implicitly represented only by whether conversion declines after cycling.

The aforementioned studies indicate that the modulation of electronic states and acidic sites constitutes a conditional optimization strategy. An explanation based on electronic effects is most compelling when experimental evidences, such as the structure of Ru under working conditions, the initial reaction rate normalized to active sites, and the electronic state, exhibit synergistic changes, while Ru dispersion and mass transfer remain constant. Similarly, the L/B ratio is meaningful only when derived from data using comparable acidic site probes and a fixed acid strength range. It cannot serve as a basis for quantitative comparison across supports with distinct properties. The proposed reaction mechanism should be validated through the following approaches, that is, comparing a series of catalysts where only one property is varied at a time, performing in situ characterization of Ru species and adsorbed intermediates, separately investigating the conversion of 4‐HPA or LA, and analyzing the retention of heteroatoms after the reaction. In the absence of such control experiments, electronic enrichment effects and metal‐acid synergy should not be regarded as proven causal mechanisms but rather described as plausible structure‐activity relationships.

### 3.2 Support Structure and Morphology Regulation

Section 2 highlights several effects that cannot be addressed solely by modifying the electronic structure of Ru, for example, intrapore diffusion may become rate‑determining, solvents can alter local adsorption and proton transfer, the metal‑support interface can modify elementary reaction barriers, and aqueous solvents may even degrade the support itself. Accordingly, the structural and morphological features of the support should be considered only when (a) stirring, particle‑size, or flow‑rate tests reveal mass‑transport sensitivity, (b) adsorption measurements indicate local enrichment, (c) interfacial structures are identified, or (d) deactivation analysis shows support decomposition or Ru migration. Support optimization strategies then include pore morphology, confinement, defects, wettability, and strong metal‑support interactions, but each single strategy often changes multiple characterization parameters at once. Importantly, claims of interfacial electronic effects require direct evidence of the catalyst structure under realistic reaction conditions.

The crystal structure, surface defects, and pore morphology of supports can modulate the Ru valence distribution and H^*^ spillover pathways through the strong metal−support interaction (SMSI), thereby affecting the distribution of non‐competitively adsorbed sites in Langmuir–Hinshelwood kinetics. On reducible oxide supports such as CeO_2_, surface oxygen vacancies anchor Ru nanoclusters and tune their electronic structure. Support acidity and basicity also participate in the dehydration‐cyclization step of the LA to GVL reaction. For instance, Gao et al. prepared ultrathin TiO_2_ nanosheet‐supported Ru catalysts, enhancing metal–support charge interaction through exposed high‐activity (101) facets, and achieved efficient LA‐to‐GVL conversion under 4 MPa H_2_ [23]. Al‐khawlani et al. prepared Ru/SSZ‐39 catalyst using SSZ‐39 zeolite with AEI topological structure as support [95]. The 8‐membered ring (8‐MR) pore channels provide mass‐transport pathways while effectively suppressing deep ring‐opening of GVL, delivering GVL selectivity of 98.3%. One‐pot synthesis introduced a mesoporous structure that partially converted BAS to LAS, cooperatively driving 4‐HPA dehydration‐lactonization. The excellent hydrothermal stability of the SSZ‐39 zeolite framework preserved structural integrity after six catalytic cycles. Yu et al. used hollow carbon spheres (HCSs) as nanoreactors and, through controlled variable studies, revealed the cavity confinement effect as a cooperative competition among electronic metal–support interaction (EMSI), reactant enrichment, and diffusion [75]. The strain in the curved carbon shell raises the Ru^0^ binding energy for inner‐loaded Ru above that of outer‐loaded Ru. Substrate enrichment within the cavity dominates, and inner‐loaded Ru@HCSs‐m achieved a hydrogenation rate of 280.37 mol_LA_ mol_Ru_^−1^ h^−1^, exceeding outer‐loaded catalyst (201.34 mol_LA_ mol_Ru_^−1^ h^−1^). The intermediate‐curvature catalyst reached optimal balance between enrichment and diffusion, with an apparent activation energy of 60.66 kJ mol^−1^, lower than the commercial Ru/C (68.10 kJ mol^−1^) (Figure 7b,c).

Li et al. loaded Ru onto hierarchical porous N‐doped carbon nanospheres (HPNC) (Figure 8a,b) [96]. GVL yield exceeded 99% in 2 h without solvent, and the catalyst is stable over six cycles. Gao et al. tuned oxygen vacancy density by varying Ru loading on LaMnO_x_ support [97]. XPS and DFT confirm that oxygen vacancies enhance LA adsorption energy. Ru_3_LaMnO_x_ produced 2‐butanol as the major product at 99.1% yield. This study sets a reference baseline for quantifying adsorption energy at defect sites. Sorokina et al. used hyperbranched polymers as support [98]. High branching density nanocavities lock Ru nanoparticles (NPs) at branch termini, steric shielding suppresses sintering, and flexible pore channels facilitate substrate diffusion. As a result, the system delivered a GVL yield of 87% with four‐cycle stability. Leguizamón‐Aparicio et al. prepared Ru catalysts on ZrO_2_‐modified Al_2_O_3_ composite supports [99]. ZrO_2_ increases Lewis acid‐site density and suppresses hydrothermal dissolution‐deactivation of Al_2_O_3_, delivering high LA conversion (99%) and GVL selectivity (99.99%). Yang et al. used organic amine‐modified vermiculite as support for Ru [100]. Amine modification widens interlayer spacing and provides coordination anchoring sites for Ru, reducing metal leaching tendency. Yang et al. reported EL hydrogenation to GVL over Ru/SBA‐15 at 25°C and 0.5 MPa H_2_ [101]. The uniform ∼7 nm mesopores of SBA‐15 anchor highly dispersed Ru of approximately 2.1 nm, and the microenvironment enrichment effect extends the operating temperature to room temperature. Vyskočilová et al. prepared Ru catalysts on natural montmorillonite (Ru/MMT), introducing solvent donor number (DN) as a quantitative descriptor for solvent control of EL hydrogenation vs. esterification selectivity [102]. High‐DN branched alcohols stabilize 4‐HPA and promote lactonization. Low‐DN linear alcohols increase esterification by‐product proportion. Ru/MMT achieved 100% GVL selectivity in a non‐protonic solvent at 120°C and 4.0 MPa of H_2_. The reaction rate was positively correlated with the DN of the solvent, revealing DN as a quantitative selectivity parameter in the engineering of LA hydrogenation solvents.

**FIGURE 8:**
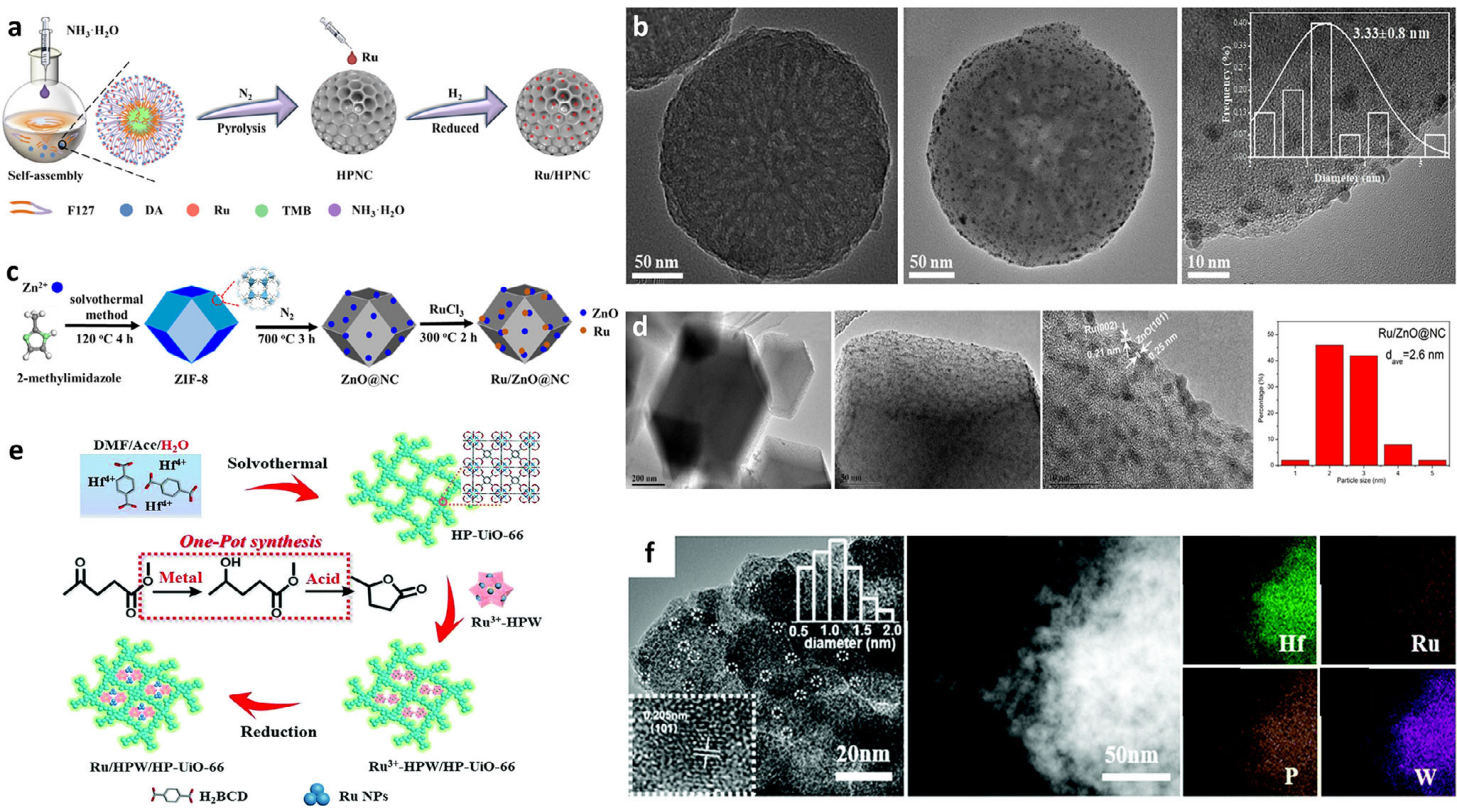
(a) Preparation procedure of Ru_x_/HPNC catalysts. (b) TEM images of the HPNC support, Ru_3_/HPNC catalyst, and histogram of Ru nanoparticle size distribution. Reproduced with permission [96]. Copyright 2022, Elsevier. (c) Preparation procedure of Ru/ZnO@NC via ZIF‐8‐derived templating. (d) TEM images of Ru/ZnO@NC and corresponding Ru nanoparticle size distribution histograms. Reproduced with permission [103]. Copyright 2024, Elsevier. (e) Schematic illustration of the synthesis of Ru/HPW/HP‐UiO‐66 by encapsulating phosphotungstic acid within hierarchically porous UiO‐66. (f) STEM image, HAADF‐STEM image, and elemental distribution maps of Hf, Ru, P, and W in Ru/HPW/HP‐UiO‐66. Reproduced with permission [104]. Copyright 2022, Royal Society of Chemistry.

Metal‐organic frameworks (MOFs)‐derived supports have yielded a series of highly active catalysts. ZIF‐8‐derived ZnO@NC supported Ru NPs, driven by bidirectional electron transfer at the Ru–O–Zn interface, reached TOF of 6032 h^−1^ at 40°C with conversion consistently >99.7% over five cycles (Figure 8c,d) [103]. Xu et al. encapsulated phosphotungstic acid within hierarchically porous UiO‐66 and prepared Ru/HPW/HP‐UiO‐66 catalyst [104]. Phosphotungstic acid tuned the intrinsic activity of Ru while hierarchical pore channels enhanced mass transfer, delivering 97% GVL yield in the tandem hydrogenation of methyl levulinate to GVL with stability exceeding seven cycles (Figure 8e,f). In La‐MOF‐derived Ru/LaQS catalyst developed by Dao et al. [46], conjugated quinoline rings anchor Ru NPs via π‐bonding. In situ CO‐DRIFT‐IR confirms that Ru^3+^ is the active site for EL activation, and Ru^0^–Ru^3+^ synergy drove EL conversion and GVL selectivity both to 99.9% at 100°C, with the catalyst maintaining stable performance over three cycles in a separate test at 90°C. Yang et al. loaded RuPt bimetallic NPs onto MIL‐101(Cr) and obtained GVL yield of 99% and TOF of 1480.5 h^−1^ at 25°C and 1 MPa H_2_ [105]. XPS confirms that the electron transfer from Pt to Ru shifts the Ru *d*‐band center and enhances C═O activation. The Cr nodes of MIL‐101 provide Lewis acid sites that cooperatively drive 4‐HPA dehydration‐cyclization, extending the heterogeneous LA hydrogenation operating temperature to room temperature.

As an alternative to crystalline MOF‐derived supports, organic–inorganic scaffolds offer distinct structural advantages, including metal‐anchoring capability, tunable acid–base functionalities, and localized solvent organization. They prove particularly valuable when evidences point to metal–acid proximity, site retention, or local adsorption effects, rather than bulk hydrogen‐donor availability, as the primary drivers of reactivity. In catalytic systems based on polymeric ionic‐liquid‐stabilized Ru NPs, Ru‐substituted polyoxometalate clusters, and polyoxometalate–ethylenediamine–Ru assemblies [106, 107, 108], the hybrid scaffolds provide an acid–base environment, while Ru performs hydrogenation. Zhou et al. coordinated Zr^4+^ with the –SO_3_H and –OH groups of biomass‐derived lignosulfonate [109]. The polymer binds the metal and also supplies Brønsted acid sites. GVL yield from LA reached about 92.5% at 150°C, with stable activity over five to six cycles. Cai et al. used the Hf‐MOF DUT‐67, in which Hf^4+^–O Lewis acid–base pairs act as the sites for alcohol‐donor reduction [110]. EL gave a high GVL yield at 160°C in isopropanol. Wu et al. anchored single‐atom Cu at linker‐defect vacancies of the Zr‐MOF UiO‐66‐(COOH)_2_ [111]. The Zr framework supplies the host and the acid sites, and the isolated Cu atoms perform the hydrogenation. In isopropanol, LA conversion was near complete and GVL yield reached 96.7% at 160°C. The Zr‐, Hf‐, and Cu‐based examples are included as architectural comparisons rather than as Ru‐specific performance benchmarks.

Several limitations of MOF‐derived support strategies deserve attention. First, the stability of MOF‐derived supports under hydrothermal acidic conditions has not been sufficiently validated. MIL‐101, UiO‐66, and related frameworks perform well in short‐term batch reactions, but most reported recycling tests are limited to 3–8 cycles, corresponding to approximately 20–50 h. Such tests are insufficient to establish long‐term structural retention under process‐relevant conditions. Progressive hydrolysis of coordination bonds in hydrothermal acidic media remains a potential deactivation pathway that has rarely been quantified over extended operation. Second, scalable synthesis routes for complex hierarchical structures, including hollow carbon spheres, yolk–shell architectures, and MOF‐derived materials, remain immature. Beyond milligram‐to‐gram‐scale preparation, these morphologies face challenges in catalyst shaping, mechanical stability, and continuous fixed‐bed operation. Consequently, the diffusion advantages observed in stirred batch reactors may not transfer directly to shaped fixed‐bed catalysts, where pressure drop and external and internal mass transfer must also be considered. Third, the activity enhancement attributed to cavity confinement is difficult to distinguish quantitatively from increases in specific surface area, changes in Ru dispersion, surface chemistry, and local adsorption. Higher surface area or confinement does not by itself demonstrate improved mass transfer. Claims of transfer enhancement should be supported by particle‐size or flow‐rate perturbations, that is, the Weisz–Prater analysis. Support morphology regulation should be considered when transfer tests, adsorption measurements, or deactivation analysis identify diffusion, local concentration, support decomposition, or metal migration as a limiting factor. The near‐term priority should therefore shift from pursuing extreme TOF values toward validating hydrothermal structural retention and engineering performance. Continuous‐flow testing, together with pressure‐drop evaluation in shaped catalysts, provides more relevant process information than additional short batch‐recycling tests of catalyst powders.

### 3.3 Particle Size and Dispersion Regulation

Ru particle size and dispersion directly determine the number and nature of accessible active sites. The mechanistic analysis in Section 2.1 established that H_2_ dissociation on Ru nanoclusters carries a negligibly low barrier, whereas the rate‐determining step of LA hydrogenation is highly sensitive to metal‐site density and coordinative unsaturation. The L−H kinetic analysis in Section 2.2 further shows that LA adsorption at acid/interfacial sites and H_2_ dissociation at Ru sites are non‐competitive. Maximizing Ru atomic utilization and optimizing interfacial sites are therefore two independent yet equally important optimization dimensions, with an inherent tension between them, that is, the high TOF of ultrasmall particles coexists with leaching risk under hydrothermal conditions. Particle size control should be prioritized only when reactant accessibility to Ru sites or Ru particle size limits the intrinsic activity. However, under continuous or practical feed conditions, Ru retention may instead become the decisive factor.

The active sites on Ru‐based catalysts span a continuous spectrum from single atoms to nanoclusters to nanoparticles, with fundamentally different catalytic mechanisms across scales. Single‐atom Ru offers 100% theoretical atomic utilization. Its unique coordination environment confers electronic structure and selectivity distinct from conventional nanoparticles. Dispersing Ru atoms on TiO_2_, MOFs, or heteroatom‐doped porous carbons has enabled efficient hydrogenation in several studies. However, single‐atom sites may face intrinsic activity limitations in elementary steps requiring adjacent metal sites for H_2_ heterolytic activation, restricting universality across reaction pathways. Shao et al. designed a RuCo single‐atom alloy catalyst that achieves high activity and GVL selectivity in aqueous‐phase LA hydrogenation through precise coordination environment control, providing direct experimental support for single‐atom catalytic strategies [25].

For the complex tandem LA to GVL reaction, the optimal architecture does not simply pursue minimum particle size. It depends on a subtle combination of factors including particle size, support properties, metal oxidation state, and metal‐support interaction [19]. The carbonyl hydrogenation step requires sufficient metal active area, while lactonization‐cyclization relies more on cooperative acid sites at the metal–support interface. Overall, Ru nanoclusters near 1–2 nm have shown favorable activity in several model‐feed systems, but an optimum size cannot yet be generalized across different supports, reaction regimes, or realistic biomass streams [75, 112]. Xu et al. kinetically revealed the competition between active‐site density and interfacial effects in Pd‐modified Ru/TiO_2_, supporting this conclusion [24].

Zhang et al. constructed Ru single‐atom and nanoparticle dual‐site catalysts on carbon fiber membranes [113]. Single‐atom Ru–N_4_ sites dramatically lower the LA to 4‐HPA hydrogenation barrier, and Ru NPs efficiently dissociate H_2_ and supply active H^*^ to neighboring single‐atom sites via spillover, so the two site types divide the catalytic labor spatially (Figure 9a–c). Han et al. used WO_x_ nanoclusters as anchoring sites to precisely disperse single‐atom Ru on N‐doped carbon (Figure 9h,i) [78]. Zhang et al. used a ZIF‐8‐derived MOF template and high‐temperature pyrolysis to prepare single‐atom Ru on TiO_2_@N‐doped carbon core‐shell supports at 0.23 wt% Ru loading [114]. AC HAADF‐STEM confirmed no agglomerated particles. XANES and EXAFS confirmed Ru–N_4_ coordination with highly unsaturated geometry. The TiO_2_ core enhances Ru–N_4_ electron density through SMSI, and DFT shows that this coordination substantially lowers the LA hydrogenation barrier. Ru_1_/TiO_2_@NC achieved efficient GVL production (100%) at room temperature and 4−6 MPa H_2_.

**FIGURE 9:**
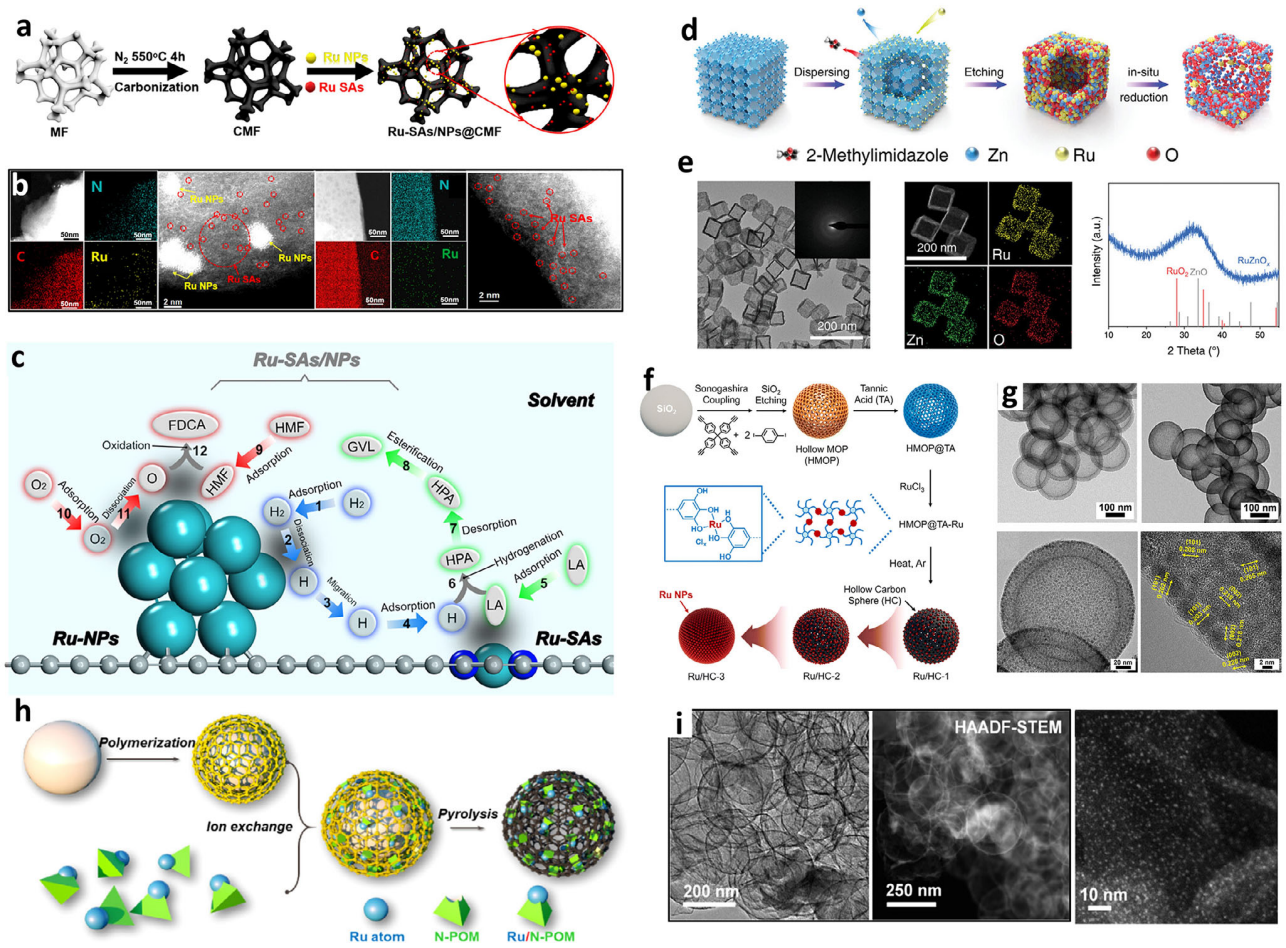
(a) Schematic illustration of the synthesis of dual‐site Ru‐SAs/NPs@CMF catalyst. (b) HAADF‐STEM images and elemental distribution maps of Ru‐SAs/NPs@CMF and the single‐atom‐only reference catalyst, confirming the coexistence of Ru single atoms and nanoparticles. (c) Proposed synergistic catalytic mechanism for hydrogenation of LA to GVL and oxidation of 5‐HMF to 2,5‐furandicarboxylic acid (FDCA). Reproduced with permission [113]. Copyright 2023, American Chemical Society. (d) Schematic synthesis of RuZnO_x_ nanoboxes. (e) TEM image, HRTEM image, atomic‐resolution EDX elemental maps, and XRD pattern of RuZnO_x_ nanoboxes. Reproduced with permission [33]. Copyright 2024, Wiley. (f) Synthetic scheme for Ru/HCS. (g) TEM and HRTEM images of Ru/HC‐3. Reproduced with permission [119]. Copyright 2026, Royal Society of Chemistry. (h) Schematic illustration of the synthesis of Ru_1_@WO_x_/CN. (i) TEM image and aberration‐corrected HAADF‐STEM image of Ru_1_@WO_x_/CN confirming atomically dispersed Ru. Reproduced with permission [78]. Copyright 2021, American Chemical Society.

Wang et al. used ZnO_x_ nanocages as templates to prepare coordinatively unsaturated Ru clusters of approximately 1 nm via structural evolution from RuZnO_x_ nanoboxes to porous nanocages [33]. The TOF reached 59 400 h^−1^ at 150°C, 30‐fold that of Ru/C, and Ru leaching was only 0.01 wt% after nine cycles (Figure 9d,e). The ultralow‐loading Ru_0.18_/Al_2_O_3_/NC achieved TOF up to 26 400 h^−1^ at 0.5 h (kinetic regime) and 99.7% GVL yield at full conversion (3 h) under solvent‐free conditions, with cumulative turnover number (TON) exceeding 10^6^ over 22 cycles (single‐batch maximum 1.77 × 10^5^) [115]. Ru loading decreased from 0.18 to 0.13 wt% across 22 cycles (∼28% leaching), with the spent catalyst still delivering 91.8% GVL yield in cycle 22.

The size–activity relationship is not monotonically decreasing. Identifying optimal size requires systematic control studies. Lan et al. prepared surface‐modified nanodiamond‐supported Ru catalysts spanning 1.2–9.0 nm and found that the TOF peaked at about 1.5 nm [116]. Further size reduction increases the C═O activation barrier due to over‐modulation by strong EMSI with the curved carbon surface. This matches the study of Cao et al. on Ru/C and Ru/Al_2_O_3_ [112], attributing the highest activity to maximal B5‐type low‐coordination site formation probability at ∼1.5 nm. These sites cooperatively accomplish H_2_ heterolytic activation and LA carbonyl activation. Meanwhile, carbon‐supported Ru^0^ has higher electron density than Al_2_O_3_‐supported Ru^0^, and K promoter further enhances activity through *d*‐band electron transfer by 2.5‐fold on carbon and sixfold on Al_2_O_3_. Guan et al. deposited RuCo alloy NPs on N‐doped carbon nanosheets, and revealed that when the NPs are completely encapsulated by the carbon layer, performance falls below that of larger exposed catalysts [117]. Wen et al. adopted CO_2_‐assisted one‐pot synthesis to fabricate Ru@CN catalyst, and Ru sites are sufficiently exposed without significantly increasing particle size, raising TOF to 28 449 h^−1^ with no activity loss after ten cycles [118]. Kong et al. prepared a high‐loading Ru/HCS catalyst with 51.6 wt% Ru loading via confined pyrolysis on hollow carbon sphere inner walls [119]. The results show that even at 51.6 wt%, Ru NPs of approximately 4 nm remain uniformly dispersed without severe agglomeration. H_2_ temperature‐programmed reduction (H_2_‐TPR) reveals that tannic acid‐derived oxygen‐containing groups provide multi‐point anchoring sites, suppressing sintering. At 100°C and 0.5 MPa H_2_, Ru/HCS achieved complete LA conversion, 94% GVL yield, and TOF of 188 h^−1^ (Figure 9f,g).

The label “single‑atom Ru” does not denote a single, uniform catalytic function. At least three structural types must be distinguished: isolated Ru atoms (with no detectable Ru–Ru coordination), sub‑nanometer clusters, and dual‑site systems comprising both single atoms and nanoparticles. Zhang et al. proposed that Ru NPs dissociate H_2_ and supply H^*^ to adjacent Ru–N_4_ sites via the spillover effect [113], whereas atomically dispersed catalysts in other studies are considered to possess intrinsic catalytic activity [78, 114]. These perspectives are not necessarily mutually exclusive, as H_2_ activation depends on the first coordination shell, support defects, adsorbed water, Ru density, and potential structural rearrangements occurring in a H_2_ atmosphere. Furthermore, the species that remain unconfirmed because they fall below the detection limits of *ex‑situ* microscopy techniques may also contribute to catalytic activity.

To demonstrate that isolated Ru atoms can independently activate H_2_, the following conditions must be met, that is, the absence of Ru–Ru coordination under reaction conditions, a positive correlation between activity and isolated site density, and the observation of direct H_2_/D_2_ exchange or kinetic isotope effects attributable to these sites. Research should integrate *in‑situ*/operando conditions and EXAFS techniques, supplemented by wavelet transform analysis, chemisorption or probe reactions, and post‑reaction microscopic characterization. Single‑atom, cluster, and dual‑site materials should not be conflated or analyzed within the same size‑versus‑activity trend. When selecting materials for engineering applications, one must also consider atom utilization efficiency, the retention rate of Ru sites under hydrothermal conditions, the cumulative GVL yield per unit of initial Ru mass, and the extent of Ru leaching. Based on the available evidence, the development of single‑atom catalysts represents a fundamental scientific challenge for elucidating site‑specific functions, rather than a proven, universal technological pathway guaranteed to ensure industrial‑scale operational longevity.

### 3.4 Reaction Conditions and Solvent Microenvironment Regulation

The L–H kinetic analysis in Section 2.2 shows that H_2_ partial pressure affects surface coverage and RDS assignment, making precise pressure control kinetically meaningful. The stronger competitive adsorption of FA than LA further indicates that the hydrogen source governs the dominant surface species and thereby influences the overall reaction rate and product distribution. Solvent polarity and protic character also affect 4‐HPA dehydration–cyclization. Water facilitates proton transfer and cyclization, whereas organic solvents alter intermediate stability through solvation effects [35]. Accordingly, reaction‐condition and solvent regulation should be prioritized when rates are coverage‐sensitive, the hydrogen‐donor pathway changes the dominant surface species, or solvent substitution alters intermediate accumulation or product distribution without changing the working‐state catalyst structure. These effects can be evaluated using pressure‐dependent initial rates, solvent isotope effects, donor balances, dissolved‐gas measurements, intermediate time profiles, and matched‐conversion controls. Coordinated optimization of the hydrogen source, solvent, and H_2_ pressure therefore provides an operational route to improving GVL yield and selectivity without modifying the catalyst structure.

The solvent effects on reaction pathway and selectivity have been systematically quantified. In CTH systems, Doherty et al. stabilized Ru NPs in situ within a polymeric ionic liquid (PIIL) matrix [106]. The unique high viscosity, low volatility, and tunable polarity of PIIL provide a stable dispersion environment for Ru NPs. PIIL also acts as a specialized solvent promoting interfacial transfer between LA substrate and active H^*^ species. The catalytic system reached GVL selectivity of 95% at LA conversion of 77% (4 h) in alcohol‐donor CTH without external H_2_. Rodiansono et al. confirmed that water molecules effectively lower the activation energy barrier through hydrogen‐bond networks [120]. The GVL yield exceeded 99% with TOF of 2730 h^−1^ at 30°C and 1.0 MPa H_2_. Van Nguyen et al. proposed an IPA/water co‐solvent strategy, finding that water substantially enhances the hydrogen‐donor activity of IPA in CTH [121]. At IPA/water of 1:1 (v/v), GVL yield improved from ∼70% in pure IPA to 93.8%. The hydrogen‐bond of water activates elimination of *β*‐H in IPA, while participating in the lactonization–dehydration equilibrium, cooperatively promoting both hydrogen transfer and cyclization.

In FA‐donor CTH, Vega Sánchez et al. used cellulose hydrolysis by‐product FA as the sole hydrogen source over a Ru‐Mg/Al mixed oxide catalyst, and found that GVL yield reached 99% at 150°C [122]. Seenivasan et al. confined Ru NPs in the gallery space of graphene oxide frameworks (Ru@GOF) [123]. The oxygen‐containing functional groups on GOF provide multiple Ru anchoring sites and impart hydrophilicity for substrate adsorption. Its 2D conjugated layered structure provides efficient electron‐transfer channels for Ru sites, improving FA dehydrogenation selectivity and preventing irreversible CO poisoning. Wan et al. anchored Ru‐substituted vanadium‐containing polyoxometalates (POA) within flexible MIL‐53(Al) pore channels, constructing a Ru‐POA/MIL‐53 bifunctional system for one‐pot CTH in FA/water system [107]. Ru substitution of V sites raises the POA oxidation potential and introduces Ru active sites. L/B acid sites in MIL‐53 pore channels drive 4‐HPA dehydration‐cyclization, and the three steps of hydrogen transfer, dehydration, and lactonization are integrated efficiently. Compared with conventional Ru/C, Ru‐POA/MIL‐53 reduces Ru usage by 90%, and GVL yield reached 98.3% with 99.3% selectivity at 160°C, with good tolerance to cellulose hydrolysis crude liquors. Peng et al. prepared polyoxometalate (POM)‐supported ethylenediamine (ED)‐functionalized Ru catalysts [108]. Keggin‐type POM provides strong Brønsted acid sites, and the ED ligand stabilizes Ru NPs and constructs an acid‐based bifunctional interface with POM acid sites. When using sodium formate as hydrogen donor, HCOONa decomposes efficiently under mild conditions and circumvents the corrosivity of FA. GVL yield reached 99% at 110°C. Gong et al. showed that GVL yield increases rapidly with pressure in the 0.4–1.0 MPa range, while above 1.0 MPa, GVL yield increases slowly [124].

Continuous‐flow industrialization‐oriented research has delivered several advances with engineering reference value. Veisi et al. immobilized Ru NPs on bimodal N,S‐doped ordered mesoporous carbon (Ru@IBOMC), achieving 99% GVL yield in continuous flow at 30°C and 30 bar H_2_ in IPA as the reaction solvent, with a weight hourly space velocity (WHSV) up to 48 h^−1^ and a space–time yield (STY) of 211 g_GVL_ g_Ru_^−1^ h^−1^ [125]. Konwar et al. operated the carbon material (CNP500)‐supported Ru catalyst continuously for 52 days at 80°C–95°C and 3–5 bar of total pressure in aqueous phase, maintaining ∼100% conversion and ∼90% GVL selectivity (Figure 10a,b) [126]. Zhu et al. used neat LA feed in a fixed bed over Ru/N‐doped carbon catalyst at 80°C under atmospheric H_2_, estimating daily processing capacity of ∼620 g_LA_ per day [90]. Rivadeneira‐Mendoza et al. systematically examined temperature effects on product distribution over Ru/hydrochar in a continuous‐flow reactor, and found that low temperature favors GVL production while high temperature promotes further conversion to valeric acid and pentenyl products, consistent with the reaction mechanism elaborated in Section 2.1 and enabling multi‐product co‐production via temperature control [127]. These studies show that long‐duration operation is possible for selected Ru catalysts under specified model‐feed conditions. In particular, Ru/CNP500 was operated for approximately 1250 h under an aqueous feed while temperature, pressure, and space velocity were varied [126]. This single demonstration should not be generalized to Ru catalysts as a class or to real biomass hydrolysates. The relevant transferable outputs are the full time‐on‐stream profile, feed composition, space velocity, cumulative GVL production, metal loss, and spent‐catalyst structure.

**FIGURE 10:**
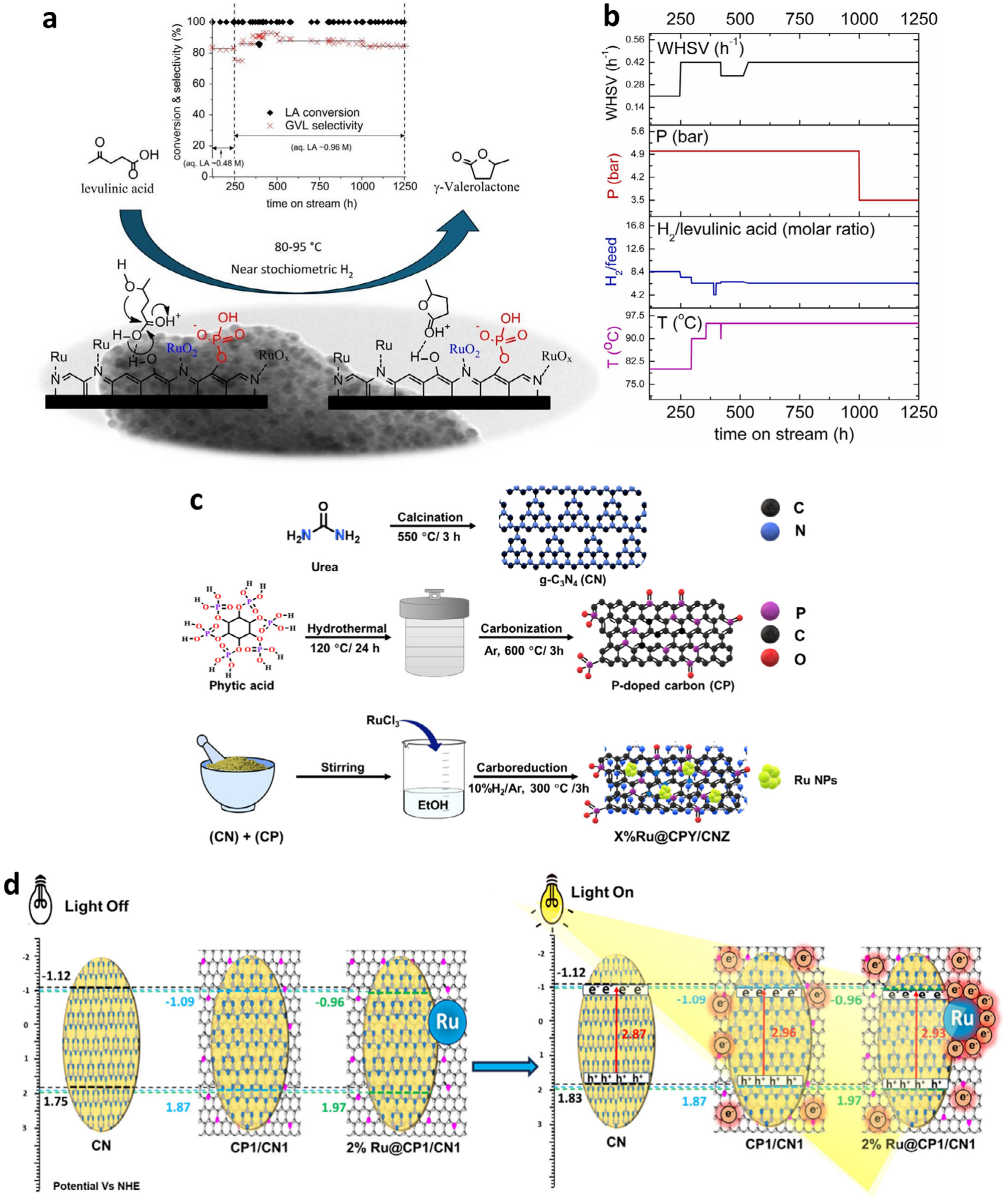
(a,b) Catalytic performance of Ru/CNP500 (5 wt% Ru) in continuous hydrogenation of aqueous LA over a 52‐day time‐on‐stream under varying reaction conditions (80°C–95°C, 3–5 bar). Reproduced with permission [126]. Copyright 2025, Elsevier. (c) Synthesis protocol for Ru@CP/CN photocatalysts with varying CP and CN weight ratios. (d) Schematic representation of the band structure of the photocatalyst under dark condition and proposed charge‐migration mechanism under visible‐light exposure, as established by in situ XPS and ultraviolet photoelectron spectroscopy (UPS). Photogenerated electrons accumulate at Ru sites via the P‐doped carbon electron‐transport channel, enhancing H_2_ dissociation. Reproduced with permission [128]. Copyright 2025, American Chemical Society.

Beyond conventional thermocatalysis, light‐, electricity‐, and magnetic‐induction‐assisted systems have been explored to modify interfacial activation, generate reducing equivalents, supply surface hydrogen, or localize heat. These approaches differ in whether molecular H_2_ is externally supplied and should not be grouped as universally H_2_‐free routes. Gyergyek et al. loaded Ru on carbon‐coated iron oxide NPs and introduced magnetic heating in the LA to GVL process [129]. The magnetic field drives localized induction heat generation in the magnetic NPs, enabling a higher temperature (137°C) on the surface of the nanocatalyst than the bulk of the medium (85°C). Arrhenius analysis gives an apparent activation energy of only 22 kJ mol^−1^ in this mode, far below conventional heating systems, resulting in an increased rate of conversion for isopropyl levulinate to GVL [129]. The Ru‐decorated *g*‐C_3_N_4_ and phytic acid‐derived carbon composite (Ru@CP1/CN1) catalyst developed by Chauhan et al. achieved 100% LA conversion and 99% GVL selectivity under 15 W blue light‐emitting diode (LED) irradiation at 0.4 MPa H_2_ and room temperature [128]. In situ XPS confirms that illumination drives charge migration from *g*‐C_3_N_4_ to Ru active sites, and P‐doped carbon serves as an electron‐transport channel accelerating photogenerated carrier separation, reducing energy input for photocatalytic hydrogenation (Figure 10c,d). More et al. systematically investigated visible‐light‐driven LA hydrogenation over Ru/CeO_2_ [130]. Photogenerated holes participate in substrate activation, and photogenerated electrons accumulate at Ru sites through metal–semiconductor charge transfer, enhancing H_2_ dissociation‐activation capability. Meanwhile, the SMSI effects optimize Ru–CeO_2_ interfacial coupling and suppress carrier recombination. At room temperature and 0.1 MPa H_2_, Ru/CeO_2_ achieved 81.5% LA conversion and 98.7% GVL selectivity. In contrast, the dark control experiment showed a conversion of less than 15%, indicating that light plays a significant role. To distinguish between photo‐generated electrons and photothermal effects, it is necessary to determine the yield at specific light wavelengths and conduct corresponding temperature control experiments. García et al. constructed a single‐unit photoelectrocatalytic device using TiO_2_ nanotube array photoanodes to in situ decompose water and supply active H^*^ for LA hydrogenation, obtaining 48.2% GVL yield near room temperature [131]. This serves as concept‐level validation of a solar‐driven, fossil‐H_2_‐free biomass refinery route.

Externally assisted systems should be classified by hydrogen‐supply mode and dominant energy input, rather than by the presence of light or electricity alone. At least five categories can be distinguished: (i) In photo‐assisted H_2_ thermocatalysis, molecular H_2_ is provided externally, and light irradiation alters the electronic states or surface chemistry of the catalyst. (ii) In photocatalytic in situ hydrogen production systems, light irradiation generates a reducing equivalent or H_2_ from water or hydrogen donors, which is then used for the reduction of LA or levulinates. (iii) Photoelectrocatalysis combines light irradiation and applied voltage. (iv) Electrocatalytic hydrogenation directly generates surface hydrogen via the reduction of protons or water at the cathode, without the need for molecular H_2_. (v) Photothermal and magneto‐induction systems primarily alter the local temperature of the catalyst. The fifth category should be compared in a dark environment matched to the reaction temperature or in a conventionally heated environment.

For photo‐assisted H_2_ thermocatalysis, light does not necessarily replace H_2_ activation or introduce a completely different reaction pathway. Instead, it can change charge distribution at the Ru‐support interface and thereby influence H_2_ dissociation and carbonyl activation. In the reported Ru_1_/CeO_2_ system, the CeO_2_ conduction‐band edge was located at approximately −0.60 V vs. NHE, which was proposed to favor electron transfer from the illuminated support to Ru sites [130]. The resulting increase in electron density at Ru was associated with easier H_2_ activation and C═O hydrogenation. A Schottky junction at the Ru–CeO_2_ interface may promote charge separation, while strong metal–support interactions may stabilize the interface and suppress electron–hole recombination. The CeO_2_ valence‐band position also indicates that photogenerated holes may participate in oxidation reactions involving LA or reaction intermediates. Such hole‐driven reactions could either assist coupled redox processes or generate undesired side products, and therefore require direct product and carbon‐balance analysis. These electronic and interfacial effects have been proposed for specific Ru/support systems and should not be generalized without wavelength‐dependent measurements, dark controls, and working‐state characterization.

The high‐yield Ru@CP1/CN1 and Ru/CeO_2_ systems should be described as photo‐assisted H_2_ thermocatalytic systems because external molecular H_2_ was supplied. Ru@CP1/CN1 gave 100% LA conversion and 99% GVL selectivity under illumination [128], whereas Ru/CeO_2_ reached 81.5% conversion and 98.7% selectivity [130]. These high‐yield results demonstrate that no general upper limit on GVL yield can be assigned to light‐assisted systems. However, high endpoint conversion or selectivity does not by itself establish high photon efficiency or energy efficiency. Photon flux, illuminated area and volume, external H_2_ consumption, reactor temperature, and quantum efficiency were not reported on a common basis, so these systems cannot yet be quantitatively ranked against one another or against conventional thermocatalysis.

Electrocatalytic hydrogenation follows a different operating principle. Active hydrogen is generated at the cathode through proton or water reduction, and the applied potential controls the surface H^*^ coverage. Increasing the cathodic potential can accelerate carbonyl hydrogenation, but an excessively negative potential promotes H^*^ recombination and hydrogen evolution, which lowers the Faradaic efficiency. Selective LA hydrogenation therefore requires a potential range that provides sufficient surface hydrogen while limiting H_2_ evolution. Metals with relatively weak hydrogen binding, including Pb, In, and Cd, can favor substrate hydrogenation over hydrogen release under selected conditions. Vilariño et al. deposited a trimetallic CuNiRu catalyst on vertically aligned graphene nanowalls and obtained 96.6% LA conversion, 92.4% GVL yield, and 98.5% GVL selectivity at 50°C, with a Faradaic efficiency of up to 89% and reported metal leaching below 1% [132]. At 5°C, 4‐HPA became the main detected intermediate, illustrating that temperature can alter intermediate accumulation and pathway balance. A partially oxidized PbS electrode using water as the hydrogen source produced GVL as the only reported product with a Faradaic efficiency of 78.6% [133].

In Ru‐containing electrocatalysts, Ru may contribute to proton reduction and surface‐H^*^ formation, while oxidized Ru species or adjacent LAS may assist the activation and lactonization of oxygen‐containing intermediates. This interpretation is consistent with proposed Ru^3+^‐assisted pathways for levulinate‐esters hydrogenation [46], but the specific functions of metallic and oxidized Ru should be verified under operating conditions. Single‐metal Ru electrodes remain uncommon, and Ru is more often used as a component of multimetallic electrodes. Direct comparison with thermocatalytic Ru catalysts is therefore difficult because the active‐site basis, electrical energy input, Faradaic efficiency, and hydrogen source differ.

Magnetic‐induction operation is better described as localized heating than as a distinct catalytic pathway unless a non‐thermal field effect is demonstrated. In these systems, a radiofrequency alternating field causes magnetic relaxation and hysteresis losses in an iron‐oxide‐containing component, generating heat within or near the catalyst particle. The catalyst surface may therefore be hotter than the measured bulk liquid. An Arrhenius analysis based only on the bulk temperature can consequently underestimate the local reaction temperature and give an apparently low activation energy. Because the magnetic energy available at an individual active site is much smaller than the energy required for C═O hydrogenation, the observed rate enhancement is more reasonably attributed to nanoscale heating unless temperature‐matched controls demonstrate an additional field effect.

These external‐field approaches therefore address different limitations. Photo‐assisted H_2_ thermocatalysis can modify charge density and interfacial activation while still relying on externally supplied H_2_. Photocatalytic in situ hydrogen‐generation systems couple light‐driven reducing‐equivalent formation with hydrogenation. Electrocatalytic hydrogenation supplies surface H^*^ without pressurized H_2_ but must suppress the competing hydrogen‐evolution reaction. Magnetic induction mainly accelerates thermal steps through localized heating, while product selectivity remains governed by the intrinsic metal–acid and adsorption properties of the catalyst.

Endpoint GVL yield, TOF, and cycle number are not sufficient for comparison across these categories. Photo‐assisted and photocatalytic studies should report wavelength distribution, incident photon flux, illuminated area and volume, temperature‐matched dark controls, apparent or external quantum efficiency, hydrogen or donor consumption, carbon balance, and stability under illumination. Electrocatalytic and photoelectrocatalytic studies should additionally report cell voltage, current density, Faradaic efficiency, electrical energy consumption per mass of GVL, and the fraction of current consumed by hydrogen evolution. Until these parameters are reported consistently, the available studies demonstrate light‐assisted enhancement, in situ photochemical or electrochemical hydrogen supply, and localized heating in selected systems, but they do not support a general efficiency ranking against thermocatalysis. These approaches should therefore be regarded as emerging alternatives or auxiliary activation methods whose practical value depends on energy efficiency, catalyst stability, hydrogen input, and integration with the intended reactor process.

### 3.5 Deactivation Resistance and Regenerability Regulation

Sections 2.1–2.3 show that the operating regime also determines the stresses imposed on the catalyst. These stresses include (i) hydrothermal leaching and support transformation favored by aqueous acidic media, (ii) increased risk of GVL deep conversion and condensation under high temperature and prolonged residence time, (iii) Ru poisoning by FA‑derived CO, and (iv) restructuring or migration of highly dispersed Ru species under reaction conditions. Therefore, deactivation control should be treated as a primary intervention when time‑dependent activity loss, Ru dissolution, particle growth, support transformation, or pore blockage is observed. Ultrasmall Ru NPs, despite their extremely high initial TOF, are more susceptible to dissolution–leaching in acidic hydrothermal environments. Practical system design must carefully balance ultrahigh dispersion‐derived activity gains against structural stability in long‐cycle operation [19]. Researchers have developed multi‐dimensional cooperative regulation strategies to address each, such as spatial confinement, bimetallic synergy, and metal‐support interaction engineering. Spatial confinement is one of the most effective current anti‐sintering strategies, since confining Ru within porous carbon shells or silica cage structures blocks inter‐particle contact migration at high temperature, extending catalyst lifetime while maintaining high initial activity [30, 73]. Introducing a second metal to build bimetallic alloys such as Ru–Co, Ru–Zn, and Ru–Re or single‐atom alloys (SAAs) uses inter‐metal electronic compensation and *d*‐band center modulation to simultaneously suppress coke formation and maintain or enhance performance while reducing Ru loading [25]. These effects together optimize activity, stability, and cost. In continuous‐flow or realistic‐feed conditions, deactivation control may constitute the first design gate rather than a downstream optimization step.

Taking advantage of the spatial confinement, Yang et al. developed yolk‐shell Ru@hollow pyridinic‐N‐doped carbon (Ru@HNC) catalyst that constrains Ru NPs within carbon shells via cavity confinement, maintaining LA conversion above 89% after eight cycles, while commercial Ru/C dropped to 61% after just four cycles (Figure 11a–c) [73]. Cao et al. prepared semi‐embedded Ru‐Al_2_O_3_@CN catalyst, in which Ru NPs are semi‐covered by N‐doped carbon layers, protects Al_2_O_3_ from hydrothermal hydrolysis on one hand and suppresses Ru leaching through strong SMSI on the other hand [134]. An activation effect emerged in early cycles, with conversion progressively rising from 61% to 72%, attributed to gradual acid‐medium corrosion of surface defect carbon layers releasing initially buried active sites, revealing a dynamic activation mechanism on defect carbon surfaces (Figure 11d,e).

**FIGURE 11:**
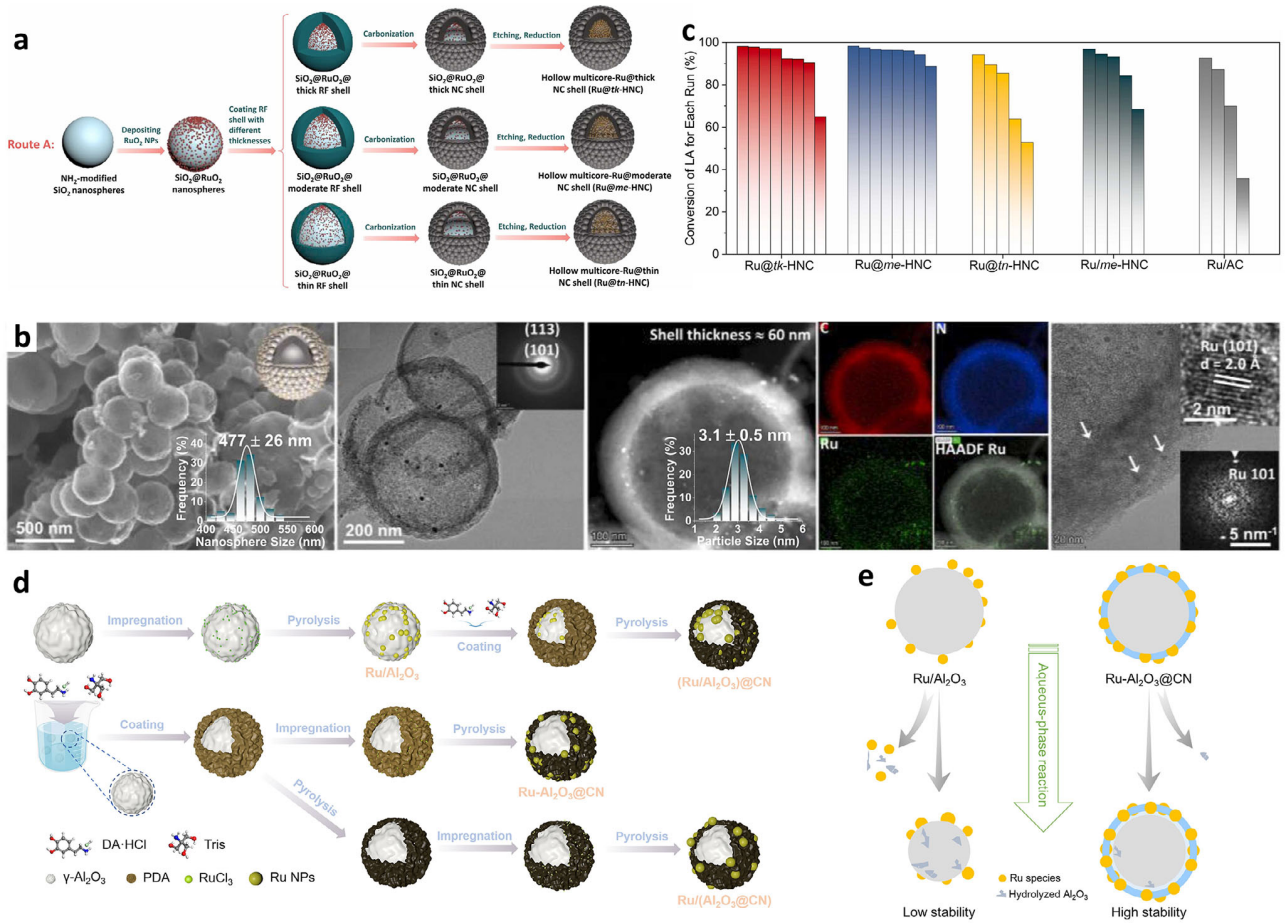
(a) Synthetic routes for yolk‐shell Ru@hollow pyridinic‐N‐doped carbon nanospheres with thick, moderate, and thin carbon shell thicknesses. (b) SEM image with schematic model and nanosphere size distribution histogram, TEM image with SAED pattern, HAADF‐STEM image with Ru particle size distribution histogram, EDX elemental mapping images, and magnified HRTEM image with FFT pattern of the Ru/*me*‐HNC catalyst. (c) Multi‐cycle stability comparison of the catalysts in LA‐to‐GVL conversion. Reproduced with permission [73]. Copyright 2024, Elsevier. (d) Schematic illustration of the synthesis procedure for Ru/Al_2_O_3_ and Ru‐Al_2_O_3_@CN catalysts. (e) Proposed stability mechanism. The semi‐embedded N‐doped carbon overlayer simultaneously shields Al_2_O_3_ from hydrothermal hydrolysis and anchors Ru NPs through enhanced metal−support interaction, suppressing Ru leaching and sintering under aqueous acidic conditions. Reproduced with permission [134]. Copyright 2024, Elsevier.

In bimetallic synergy examples, Cai et al. prepared Cu–Ru/ZrO_2_–SO_3_–SiO_2_ catalyst that maintained >95% GVL yield in EL CTH after five cycles, requiring only IPA washing between cycles [135]. XRD, TEM, and XPS confirmed unchanged active metal structure, morphology, and chemical state after cycling. Inductively coupled plasma (ICP) showed no significant metal leaching. The good stability is derived from S species in the ZrO_2_–SO_3_–SiO_2_ composite support that directionally transfer electrons to Cu and Ru, forming electron‐enriched active sites while substantially enhancing metal–support interaction to anchor metal particles. Cu–Ru bimetallic cooperative interaction suppresses leaching and sintering of single metals under hydrothermal conditions. Ding et al. deposited amorphous alloy SnRuB on sulfonated activated carbon to form Ru_2_Sn_3_/Ru_3_Sn_7_ binary alloy phases, blocking single‐Ru particle sintering–migration pathways [136]. Butyl levulinate conversion consistently exceeded 94% after six cycles at 180°C and 3 MPa H_2_. It should be noticed that the cycle numbers and leaching values reported cannot be compared strictly. They were generally obtained under different temperatures, feeds, batch or flow modes, and detection limits. A catalyst that looks more stable may simply have been tested under milder conditions.

In metal‐support interaction engineering, Liu et al. demonstrated that the strong Ru–MnO_x_ interaction in Ru–MnO_x_/Al_2_O_3_ promotes reduction of Mn^3+^ to Mn^2^^+^ and generates surface oxygen vacancies that act as Lewis acid sites and donors to Ru electron density [137]. The activity in hydrodeoxygenation of LA to 2‐butanol scaled linearly with [Mn^2^^+^]ln([Ru^0^]). At 200°C and 5 MPa H_2_, the TOF reached 1.99 × 10^6^ h^−1^ with 2‐butanol yield of 98.8% and GVL formed as the intermediate. The Ru–MnO_x_ coupling functions as a strong metal–support interaction that anchors Ru against migration under hydrothermal acidic conditions, motivating its placement among deactivation‐resistance strategies. Patil et al. used P25 TiO_2_‐supported Ru as a model catalyst to systematically study deactivation mechanism and kinetics in LA to GVL [138]. TEM confirmed Ru particle growth from 1.38 nm on the fresh catalyst to 2.38 nm on the spent catalyst. XPS confirmed partial TiO_2_ reduction, with TiO_x_ species migrating outward to encapsulate Ru, forming a classical SMSI‐induced deactivation resistance. Strong coordination adsorption of the LA carboxyl group accelerated Ru particle mobilization and sintering.

For coking suppression, the first strategy is acid‐site moderation. In Ru/NbOPO_4_/SBA‐15 catalyst, NbOPO_4_ supplies moderate, hydrothermally stable Brønsted acid sites, which sustain dehydration–cyclization without the excess strength that starts AL oligomerization [139]. The catalyst ran for 300 h in a continuous fixed‐bed reactor at 90°C–100°C and 10 bar H_2_ with stable activity and 72%–86% GVL selectivity. Thermogravimetric analysis (TGA) of the spent catalyst showed a broad desorption feature near 390°C, assigned to chemisorbed heavy oxygenates rather than graphitic coke. The second strategy is H_2_‐rich operation. Sufficient H_2_ partial pressure keeps surface H^*^ concentration high, so coke precursors are hydrogenated before they polymerize. In line with this, Guan et al. found that pore volume and Ru surface area both rose after cycling of Ru@CN at 100°C and 2 MPa H_2_, which indicates Ru surface liberation rather than coke build‐up [117].

Current anti‐deactivation research has a methodological gap, that is, virtually all stability tests are conducted in batch mode with neat LA aqueous solution, while real industrial scenarios require catalysts to operate long‐term in continuous‐flow fixed‐bed or slurry reactors with complex feed streams containing furfural, organic acids, metal ions, and other impurities. An undeniable testing‐condition gap separates existing batch cycling data from industrial catalyst lifetime standards. Direct extrapolation from batch cycling to industrial stability carries major uncertainty. Ru leaching detection limit differences across studies may render “not detected” conclusion non‐comparable. Establishing a standardized evaluation protocol covering true hydrothermal conditions, multi‐cycle long‐duration continuous testing, quantitative metal leaching analysis, and catalyst structural integrity characterization is an urgent priority for advancing field consensus. The absence of a unified evaluation protocol is a key methodological shortcoming in current anti‐deactivation research. Adopting industrial catalyst lifetime evaluation conventions as baseline testing conditions should take priority over the pursuit of single‐cycle performance metrics.

The stability of a catalyst is a variable that changes with experimental conditions. The number of cycles holds practical significance only when factors such as total cycle duration, catalyst recovery, regeneration method, feed composition, and cumulative substrate conversion are comprehensively considered. Reports on stability should include, at a minimum, the total operating time, the cumulative GVL yield calculated based on the initial mass of Ru in the catalyst, the productivity over time, the quantitative analysis of Ru leaching, the changes in Ru particle size and support phase, and the regeneration procedures. Following simulated feed testing, experiments assessing the impact of impurities and continuous operation tests using actual substrate should be conducted. Rather than simply stating that “no leached Ru was detected,” the upper limit of the value should be reported based on the detection limit of the analytical method used.

### 3.6 Criteria for Selecting Regulation Strategies

The five catalyst design strategies mentioned above aim to enhance catalyst performance. When selecting an appropriate strategy, the reaction system must be considered first. Factors such as substrate type, hydrogen source, reaction phase, reactor type, and feed composition determine the comparability of different research results. A high TOF does not guarantee catalyst longevity, while activity measured under mass‐transfer‐limited conditions fails to accurately reflect the intrinsic activity.

For the low‐temperature liquid‐phase hydrogenation of LA using external H_2_, it is essential to first verify that the measured rate is not limited by mass transfer. The effects of stirring speed and catalyst particle size should be investigated, and gas‐liquid mass transfer as well as intraparticle diffusion effects must be evaluated. Once heat‐ and mass‐transfer limitations have been excluded, a positive H_2_ reaction order over a specified pressure range together with a reproducible H_2_/D_2_ kinetic isotope effect supports a contribution from H_2_ activation or hydrogen transfer to rate control under those conditions. These observations do not identify a unique rate‐determining step or distinguish H_2_ dissociation from subsequent hydrogen transfer. In this scenario, relevant engineering aspects include the number of accessible Ru active site clusters, the coordination environment of Ru, and the electronic state of Ru under reaction conditions. If the reaction rate shows weak dependence on H_2_ pressure and is accompanied by the accumulation of 4‐HPA, the conversion of 4‐HPA must be assessed independently. Subsequently, selective modification or poisoning of acidic sites can be employed to determine whether the cyclization of 4‐HPA is the key step limiting GVL formation. If cyclization is kinetically critical, then acidic site density, acid strength, and the distance between metal and acidic sites become important design parameters. Dependence on H_2_ pressure or the accumulation of 4‐HPA alone is insufficient to establish a universally applicable RDS.

For transfer hydrogenation reactions using FA, the competitive adsorption behavior between FA and LA, the two decomposition pathways of FA, and the surface CO coverage should be clarified before altering Ru particle size or electronic state. The decomposition pathways of FA include dehydrogenation (yielding H_2_ and CO_2_) and dehydration (yielding CO and H_2_O). Consequently, key performance indicators involve selectivity toward the dehydrogenation pathway, surface CO coverage, the FA/LA molar ratio, and the dependence of LA conversion on FA concentration. If a catalyst promotes CO formation and leads to CO poisoning, the GVL yield may decrease, even if the catalyst accelerates FA conversion. For alcohol‐mediated transfer hydrogenation, isotope labelling experiments should be employed to distinguish between processes such as alcohol dehydrogenation, esterification or transesterification, hydrogen transfer, and lactonization. Compared to catalyst characterization data obtained from hydrogenation using external H_2_, factors such as the density and strength of acid‐base sites, alcohol structure, water content, and the residence time of reaction intermediates are more critical.

Levulinates must be distinguished from LA. They lack free carboxyl groups, and their conversion processes may rely more heavily on Lewis acid activation and the alcohol elimination step during lactonization. Consequently, mechanistic inferences involving Ru^3+^ species or LAS on the support cannot be directly applied to the aqueous‐phase H_2_ hydrogenation of LA. High‐temperature gas‐phase reactions may also follow different pathways and are more prone to further conversion of GVL. Under such conditions, reaction time, acid strength, and GVL stability must be strictly controlled. For continuous operations using either simulated or actual feedstocks, catalyst stability is often more important than initial activity. Key recognizable parameters involve deactivation rate, Ru leaching, structural changes in the support, pore blockage, tolerance to feedstock impurities, cumulative GVL yield per unit mass of Ru, and catalyst regenerability. These factors should be thoroughly considered before attempting to maximize Ru dispersion.

Based on the evidences provided by characterization techniques, a progressive system can be employed to identify bottlenecks in a reaction. Level I evidence comprises performance metrics such as yield and cycling data. While these reveal trends, they do not pinpoint underlying causes. Level II evidence incorporates kinetic or isotopic measurements, ruling out heat and mass transfer limitations. Level III evidence combines Level II data with characterization information regarding active sites or intermediates under reaction conditions. Level IV evidence builds upon Level III by systematically altering catalyst properties or reaction conditions, and by using kinetic or theoretical models to generate verifiable predictions that align with experimental observations. These levels reflect a progression toward greater evidentiary robustness, though this does not imply that every study must meet the Level IV standard.

These levels of evidence can guide the selection of regulation strategies by demonstrating the extent of the impact on specific bottlenecks. For instance, the observed H_2_ pressure dependence and isotope effect can be tested against changes in accessible Ru‐site density under otherwise comparable conditions. A correlation would support a role for these sites, but its absence would not exclude their involvement, because surface coverage and site accessibility may also change. If 4‐HPA accumulates and its independently measured cyclization rate correlates with the quantified properties of acidic sites, then controlled modification of these sites should alter the GVL formation rate without causing a proportional change in the initial rate of LA hydrogenation to 4‐HPA. If this response is not observed, the proposed rate‐controlling role of cyclization would be less convincing under the tested conditions. If catalyst deactivation under actual feed conditions is attributed to Ru leaching, then stronger Ru anchoring should reduce the concentration of dissolved Ru and increase the cumulative GVL yield per unit mass of Ru. If Ru leaching remains unchanged, the proposed stabilization mechanism would require further testing. Rather than predicting the optimal catalyst in advance, these guidelines specify the measurements required before selecting an optimization strategy, as well as the expected results that would support or refute a proposed explanation. Within this framework, Table 3 summarizes the mechanistic basis, principal advantages, current limitations, and cross‐strategy synergies of the five regulation strategies.

**TABLE 3:** Comprehensive comparison and relationships of five regulation strategies.

Regulation strategy	Mechanism	Advantages	Limitations	Synergy with other strategies
3.1 Electronic structure and acid site regulation	Heteroatom doping generates electron‐rich Ru^0^; Lewis/Brønsted acid‐site synergy drives dehydration–cyclization	Activation barrier substantially reduced; Acid sites are precisely tunable	Spatial distribution of heteroatoms is difficult to control; N‐C hydrothermal stability data are insufficient	With 3.3: N‐doping tunes both electronic structure and dispersion; With 3.5: N anchoring suppresses leaching
3.2 Support structure and morphology regulation	SMSI modulates Ru valence; Pore channel mass‐transfer enhancement; Cavity confinement	Mass transfer and activity co‐enhancement; Porous structure balances dispersion and stability	Hydrothermal stability of MOFs is limited; Complex structure scale‐up is difficult	With 3.1: SMSI simultaneously tunes acid sites; With 3.5: pore confinement suppresses sintering
3.3 Particle size and dispersion regulation	B5‐type site optimization; Single atom–cluster dual‐site division	Atomic utilization is maximized; H_2_ dissociation and carbonyl activation are cooperated	Long‐term stability of single‐atom Ru is insufficient; H_2_ dissociation may require neighboring sites	With 3.2: pore channels anchor and limit particle growth; With 3.1: particle size determines B5‐site density
3.4 Reaction conditions and solvent microenvironment regulation	H_2_ pressure optimization; Water–organic co‐solvent synergy; CTH hydrogen‐donor selection	Performance can be optimized without altering catalyst structure; Green hydrogen‐source integration	FA‐CTH is constrained by CO poisoning; High‐pressure H_2_ safety/cost elevated	With 3.5: optimized solvent reduces metal leaching; With 3.1: water assists acid‐site proton transfer
3.5 Deactivation resistance and regenerability regulation	Spatial confinement suppresses sintering; Carbon‐layer anchoring prevents leaching; SMSI dynamic analysis	Directly extends industrial catalyst lifetime; Directly addresses catalyst lifetime and Ru retention	Most tests remain short‐term batch; Real feed stream stability data are scarce	With 3.2: support structure protects metal from hydrothermal attack; With 3.3: appropriate particle size reduces leaching risk

Table 4 summarizes representative performance data of Ru‐based catalysts in conversion of LA and levulinates to GVL under different reaction conditions. The entries differ in substrate, hydrogen source, solvent, concentration, catalyst and Ru loading, temperature, pressure, reaction time, reactor mode, conversion, TOF definition, and durability protocol. Because the underlying articles do not report all normalization variables consistently, missing quantities cannot be reconstructed without assumptions. Accordingly, neither row order nor the reported conversion, yield, TOF, or cycle number should be interpreted as an activity or stability ranking. Cross‐study conclusions in this review are therefore based on reaction‐regime‐specific evidence discussed in the text, rather than on numerical comparison across Table 4.

**TABLE 4:** Catalytic performances of Ru‐based catalysts reported recently in conversion of LA and levulinates to GVL.

Catalyst	Ru loading (wt%)	Substrate	Hydrogen source	Temperature (°C)	Conversion (%)	GVL Yield (%)	Operating time (h)	Ru‐normalized productivity (mol_GVL_ g_Ru_^−1^ h^−1^)	TOF (h^‒1^)	Stability	Total operating time (h)	Reactor mode	Refs.
Ru/TiO_2_	0.83	LA (0.5 M)	H_2_ (4 MPa)	100	100	99.1	1.5	31.83	19 045	Yield of 96% after 6 cycles	9	Batch	[23]
RuZnO_x_	33.1	LA (0.2 M)	H_2_ (3 MPa)	150	99	99	0.0333	384.05	59 400	Yield of 92% after 10 cycles	0.7833	Batch	[33]
Ru/HZSM(40)‐5	4.2	LA (0.169 M)	H_2_ (1 MPa)	100	98	98	0.5	9.74	—	—	0.5	Batch	[40]
Ru_2_/LaQS	1.6	EL (0.125 M)	H_2_ (2 MPa)	100	99.9	99.9	6	0.20	—	Yield of 99.9% after 3 cycles	19	Batch	[46]
Ru@tn‐HNC	9.8	LA (0.5 M)	H_2_ (1 MPa)	30	94.3	94.2	3.5	0.47	18 733.4	Yield of 64% after 4 cycles	14	Batch	[73]
Ru@PN‐C	23.9	LA (0.5 M)	H_2_ (1 MPa)	30	98.5	98.4	3.5	47.05	20 339	Yield of 98% after 4 cycles	14	Batch	[74]
Ru@HCSs‐m	—	LA (0.287 M)	H_2_ (4 MPa)	180	99.07	90.37	4	3.64	—	Yield of 90% after 5 cycles	20	Batch	[75]
Ru/CNF‐ILs	0.74	LA (0.5 M)	H_2_ (1 MPa)	100	96	90	2	15.20	17 222	Yield of 80% after 5 cycles	10	Batch	[76]
Ru/UCN	2.82	LA (0.2 M)	IPA	100	100	99.8	12	0.07	—	Yield of 98.6% after 5 cycles	20	Batch	[77]
Ru1@WO_x_/CN	4.7	LA (solvent‐free)	H_2_ (2 MPa)	100	99	99	2	0.10	1060	Yield of 90% after 6 cycles	12	Batch	[78]
Ru_3_TPA	0.68	LA (4.83 M)	H_2_ (1 MPa)	130	95	95	2	0.14	—	Yield of 91% after 5 cycles	10	Batch	[82]
Pt‐Ru(1:0.25)/Yt	3	LA (0.86 M)	H_2_ (2 MPa)	160	100	97.99	6	0.47	—	Yield of 98% after 3 cycles	18	Batch	[83]
Ru/(AlO)(ZrO)_0.1_	3.96	LA (0.98 M)	H_2_ (1 MPa)	120	100	100	6	1.37	—	Yield of 14.7% after 5 cycles	30	Batch	[84]
5% Ru/Sn‐SBA‐15	4.85	LA (1 mL h^−1^)	Flowing H_2_ (25 mL min‒1)	250	99	97.02	3	—	—	Yield of 92% after 5 cycles	15	Fixed‐bed	[85]
4Ru7.5Nb	4.98	LA (0.172 M)	H_2_ (2 MPa)	70	30	29.1	2	0.20	34.2	—	2	Batch	[87]
3% Ru/HPS	3	LA (0.172 M)	H_2_ (2 MPa)	120	99	98	2	7.02	—	Yield of 81% after 3 cycles	6	Batch	[88]
Ru/LDMCN‐20%	18.4	LA (0.043 M)	IPA	110	100	99.3	3	1.55	—	Yield of 80% after 5 cycles	15	Batch	[89]
Ru/NC‐800	2	LA (solvent‐free)	H_2_ (1 MPa)	80	99	98	2	1.14	358	Yield of 98% after 5 cycles	2.5	Batch	[90]
Ru(1)/NC	0.72	LA (0.167 M)	H_2_ (1 MPa)	100	99.4	91.7	3	4.25	749	—	3	Batch	[91]
3Ru/BEA150	3	LA (0.286 M)	H_2_ (1 MPa)	120	96.5	94.37	24	0.13	—	Yield of 92% after 4 cycles	96	Batch	[92]
5Ru@FC‐30%	8.6	LA (0.195 M)	IPA	130	100	98.6	3	0.12	—	Yield of 92% after 5 cycles	25	Batch	[93]
5%MgSiO_3_@2%Ru@MFI	2	EL (0.141 M)	H_2_ (0.5 MPa)	80	100	98	2	0.69	—	Yield of 68% after 2 cycles	4	Batch	[94]
2%Ru/SSZ‐39	0.192	LA (0.861 M)	H_2_ (2 MPa)	150	98.05	95.4	4	10.70	—	Yield of 90.3% after 6 cycles	24	Batch	[95]
Ru_3_/HPNC	3	LA (solvent‐free)	H_2_ (2.5 MPa)	100	94.5	87.88	2	9.53	—	—	2	Batch	[96]
Ru‐PPP‐S	1.617	LA (0.195 M)	H_2_ (2 MPa)	100	99.9	99.9	4	—	—	Yield of 97% after 4 cycles	16	Batch	[98]
Ru3/ZA	3	LA (0.6 M)	H_2_ (3 MPa)	120	99.9	99.9	1	7.99	—	Yield of 95% after 5 cycles	5	Batch	[99]
Ru/O‐V	—	LA (0.766 M)	H_2_ (4 MPa)	130	100	99	5	—	—	Yield of 95% after 14 cycles	70	Batch	[100]
3Ru‐SBA‐15	3	EL (0.25 M)	H_2_ (0.5 MPa)	25	82	79.54	1	2.65	—	Yield of 79% after 6 cycles	6	Batch	[101]
Ru/ZnO@NC	1.8	LA (0.5 M)	H_2_ (4 MPa)	100	99.9	93.7	1	10.41	6032	Yield of 98.6% after 5 cycles	5	Batch	[103]
Ru/HPW/HP‐UiO‐66	0.83	Methyl levulinate (0.0768 M)	H_2_ (1 MPa)	80	97	96.03	12	0.30	—	Yield of 95% after 7 cycles	84	Batch	[104]
Ru_4_Pt_1_/MIL‐101	1	LA (0.5 M)	H_2_ (1 MPa)	25	99.9	99.9	6	0.83	1480.5	Yield of 95% after 8 cycles	48	Batch	[105]
Ru‐SAs/NPs@CMF	—	LA (0.2 M)	H_2_ (3 MPa)	150	99	99	4	—	20 417	Yield of 85% after 9 cycles	36	Batch	[113]
Ru/TiO_2_@CN	0.14	LA (0.5 M)	H_2_ (6 MPa)	30	99	99	13	0.03	—	Yield of 90% after 3 cycles	36	Batch	[114]
Ru_0.18_/Al_2_O_3_/NC	0.18	LA (solvent‐free)	H_2_ (4 MPa)	150	100	99.7	3	158.78	26 400	Yield of 91.8% after 22 cycles	66	Batch	[115]
Ru@CN‐8	8.3	LA (0.861 M)	H_2_ (2 MPa)	100	100	99.5	1.5	3.44	2043.8	Yield of 84.7% after 5 cycles	7.5	Batch	[117]
Ru@CN‐700‐80	1.26	LA (0.861 M)	H_2_ (1 MPa)	100	80	80	0.5	27.30	28 449	Yield of 80% after 10 cycles	5	Batch	[118]
Ru/HC‐3	51.6	LA (1 M)	H_2_ (0.5 MPa)	100	100	94	2	—	188	Yield of 93% after 5 cycles	10	Batch	[119]
Ru‐(0.55)MoO_x_/C	5	LA (0.667 M)	H_2_ (1 MPa)	30	100	99	2	0.28	2730	Yield of 70% after 10 cycles	10	Batch	[120]
Ru‐PC	5.6	LA (0.195 M)	IPA	140	100	93.8	3	0.18	—	—	—	Batch	[121]
MO‐Ru	1	LA (20 M)	FA	150	100	99	3	2.67	—	Yield of 98% after 4 cycles	4	Batch	[122]
Ru@GOF‐PDBA	5	LA (12 M)	FA	90	99	93.1	8	0.28	7240	Yield of 87.4% after 5 cycles	40	Batch	[123]
Ru@IBOMC	0.025	LA	H_2_ (3 MPa)	90	100	99	—	—	—	—	—	Fixed‐bed	[125]
MN‐C–Ru	4.5	LA (0.973 M)	H_2_ (1 MPa)	‒	100	100	1	6.98	—	Yield of 99% after 5 cycles	5	Batch	[129]
Ru‐Al_2_O_3_@CN	0.9	LA (4.306 M)	H_2_ (2 MPa)	120	54	46.98	1	0.64	28 009	Yield of 70% after 5 cycles	5	Batch	[134]
Cu–Ru/ZrO_2_–SO_3_–SiO_2_‐450	—	EL (0.2 M)	—	180	100	100	12	—	—	Yield of 95.4% after 5 cycles	60	Batch	[135]
20Sn1RuB/SAC	0.6	Butyl levulinate (0.452 M)	H_2_ (3 MPa)	180	99.2	83.12	3	1.12	3.87	Yield of 84.9% after 6 cycles	18	Batch	[136]

*Note*: Ru‐normalized productivity was calculated only for batch reactions using the equation defined above. Values were not calculated for continuous‐flow/fixed‐bed experiments or when the required data were unavailable. TOF values are reproduced from the cited literature. Because their calculation bases and active‐site definitions are not identical among studies, they should not be directly compared across different reports.

For batch reactions in Table 4, the Ru‐normalized productivity (*P*_Ru_) was calculated according to:

$$P_{Ru}=\frac{n_{GVL}}{m_{Ru}\times t}=\frac{n_{0}\times Y_{GVL}}{m_{cat}\times \omega_{Ru}\times t}$$

where *n_GVL_* is the amount of GVL produced, *n_0_* is the initial amount of LA or levulinates, *Y_GVL_* is the reported GVL yield expressed as a fraction, *m_Ru_* is the mass of Ru on the catalyst, *m_cat_* is the catalyst mass, *ω_Ru_* is the experimentally measured Ru mass fraction determined by ICP, and *t* is the batch reaction time. Ru‐normalized productivity was not calculated for continuous‐flow/fixed‐bed experiments.

## 4 Conclusions and Outlook

### 4.1 Summary

This review systematically surveys recent progress on Ru‐catalyzed hydrogenation of LA and its esters to GVL, covering reaction mechanisms, reaction engineering, catalyst regulation strategies, and the requirements for practical application. The thermodynamic and kinetic foundations of this conversion are first discussed by comparing the hydrogenation‐lactonization pathway via 4‐HPA with the dehydration‐hydrogenation pathway via AL. Reported RDS assignments and H_2_ reaction orders across different reaction systems are also briefly compared. Reaction systems using H_2_, FA, and alcohols as hydrogen sources are distinguished. On this basis, recent advances in catalyst development are summarized in terms of five interconnected regulation strategies: regulation of the electronic structures of Ru and acid‐base sites, support structure and morphology, Ru particle size and dispersion, reaction conditions and solvent microenvironment, and deactivation resistance and regenerability. The main conclusions involve the following aspects:

- The conversion of LA and levulinates to GVL generally cannot be described by a single reaction pathway or a single RDS. Low‐temperature liquid‐phase hydrogenation of LA generally proceeds through carbonyl hydrogenation to 4‐HPA followed by lactonization, whereas the AL pathway becomes dominant at higher temperatures, particularly in gas‐phase reactions. However, the dominant pathway and the RDS still depend on the substrate, catalyst, hydrogen source, solvent, pressure, temperature, and reactor type. A fitted H_2_ reaction order alone cannot identify the RDS, and a high energy barrier calculated for an idealized surface model should not be directly regarded as the RDS in an actual reactor. Meaningful comparisons require clearly defined H_2_ pressure and conversion ranges.
- LA, levulinates, and different hydrogen‐supply systems should not be assumed to follow the same reaction mechanism. Because levulinates do not contain a free carboxyl group, their conversion may depend more strongly on Lewis‐acid activation and C═O bond transformation. In FA‐mediated systems, competitive adsorption between FA and LA, the balance between FA dehydrogenation and dehydration, and CO poisoning may be more important than the overall FA conversion. In alcohol‐mediated systems, alcohol dehydrogenation, esterification or transesterification, hydrogen transfer, and lactonization are coupled and must be distinguished before assigning the RDS. Mechanistic conclusions obtained from one reaction system therefore cannot be directly applied to another.
- Catalyst performance cannot be explained by a single factor. Changing the support, introducing heteroatoms, decreasing the Ru particle size, or constructing confined structures generally alters several properties simultaneously, including the Ru electronic state, dispersion, acid‐base properties, wettability, adsorption, and diffusion. Clear trade‐offs also exist among these changes. Smaller Ru NPs can improve atomic utilization but may be more susceptible to leaching or structural reconstruction. Stronger metal‐support interactions can stabilize Ru but may also mask accessible active sites. Increasing acidity can promote 4‐HPA conversion while accelerating GVL ring opening and further conversion. Porous or confined structures can enrich reactants and suppress sintering but may also introduce diffusion limitations. Therefore, a high yield or TOF alone is insufficient to explain why a catalyst performs well or to demonstrate that its advantage can be maintained under different reaction conditions.
- At the catalytic performance regulation level, five complementary regulation strategies have emerged in general. (1) Electronic structure and acid site regulation. N/P/S doping constructs electron‐rich Ru^0^ states, and SMSI effects on reducible supports tune *d*‐band center of Ru, substantially lowering the C═O activation barrier. Dual‐acid‐site synergy can contribute to selectivity control, and a higher Lewis/Brønsted acid ratio has been associated with enhanced GVL selectivity in some catalyst systems. However, its significance depends on catalyst characteristics, acid‐site characterization, and reaction conditions. (2) Support structure and morphology regulation. Hierarchically porous structures including mesoporous carbons, modified montmorillonite, and vertically aligned graphene foams are beneficial for promoting GVL yield by enhancing mass transfer, increasing specific surface area, and improving Ru dispersion. (3) Particle size and dispersion regulation. Tuning Ru particle size and dispersion from nanoclusters to single‐atom regimes can markedly affect TOF, although this effect depends on catalyst structure, reaction conditions, and the TOF definition. Coordinatively unsaturated Ru sites can lower H‐addition barriers in specific systems, supporting structure–activity relationships. (4) Reaction conditions and solvent microenvironment regulation. Adjusting the water–organic solvent ratio, supercritical CO_2_ biphasic extraction, and optimizing substrate/hydrogen‐donor ratios in CTH systems enable fine‐tuned pathway selection and product purity control. (5) Deactivation resistance and regenerability regulation. Spatial confinement, bimetallic synergy, and metal‐support interaction engineering are well‐developed strategies to suppress Ru nanoparticle sintering, agglomeration, and leaching under hydrothermal acidic conditions, with multi‐cycle stability substantially exceeding conventional supported reference catalysts.
- The available literature does not demonstrate that Ru is always the best choice. Its higher cost can only be economically justified when its advantages in low‐temperature activity, selectivity, productivity, lifetime, recoverability, or product separation outweigh the overall benefits offered by non‐noble metal catalysts such as Ni‐, Cu‐, Co‐, or Zr‐based alternatives. Such comparisons cannot be based solely on substrate conversion and product yield. They should also include continuous operating time, cumulative GVL production per unit mass of Ru, Ru loss and recovery, impurity tolerance, H_2_ or hydrogen‐donor consumption, and separation requirements. These metrics have not yet been reported consistently enough to establish a universally applicable ranking of catalyst performance.

### 4.2 Conditional Industrial Boundary for Selecting Ru

The final GVL yield alone should not serve as the sole criterion for selecting Ru. Under given conditions (including substrate, hydrogen source, and reactor type), the factors to compare include space‑time yield, cumulative GVL production per unit mass of Ru, operating time, Ru loss and recovery rate, impurity tolerance, hydrogen or donor costs, and GVL separation difficulty. If higher temperatures or metal loadings are acceptable, Ni‑ and Cu‑based routes may be more attractive. Although Zr‑based transfer hydrogenation processes avoid the need for pressurized hydrogen, they require effective separation of the hydrogen donor. For Co‑based systems, heterogeneous and homogeneous catalytic routes must be evaluated separately. Existing reports have not provided these metrics consistently enough to allow a quantitative ranking across different processes. Accordingly, this review outlines the evaluation metrics needed to determine whether Ru is economically viable.

### 4.3 Key Challenges

The advances described above have materially driven the mechanistic understanding of Ru‐based catalysts for the conversion of LA and its esters to GVL, yet several key challenges remain unresolved on the path toward practical industrial application.

1. Enhancing Ru utilization efficiency while preserving stability under reaction conditions is essential. However, maximizing atomic dispersion is not a universal remedy, because isolated atoms, sub‑nanometer clusters, and nanoparticles differ in hydrogen activation, interfacial behavior, and adsorption characteristics. Accordingly, the goal should be to maximize the cumulative GVL yield per unit mass of initial Ru under a given set of reaction conditions, while simultaneously maintaining reactivity, regenerability, and GVL productivity. As a platform for elucidating active‑site specificity, single‑atom catalysts require confirmation of their Ru state under reaction conditions and assurance of long‑term stability to realize their industrial application value.
2. Insufficient tolerance to complex real feed streams. The vast majority of published studies use neat LA or neat levulinate esters as reaction substrates in clean solvents. Actual biomass hydrolysis liquors contain complex impurities such as furfural, hydroxymethylfurfural, humin precursors, and sulfur‐containing compounds, all of which can substantially suppress catalyst activity. Soluble lignin fragments and humin precursors rapidly foul porous supports, while strong competitive adsorption from acetic acid can neutralize the basic sites required for lactonization. Systematic studies on real feed systems are exceedingly scarce. Simply demonstrating batch activity in pure water cannot guarantee survival in actual hydrolysate streams, since simultaneous active‐site poisoning and acid‐site neutralization define the true industrial bottleneck.
3. Trilemma of activity–selectivity–stability. Raising reaction temperature accelerates 4‐HPA dehydration–cyclization but promotes GVL ring‐opening to valeric acid or over‐hydrogenation to 2‐MTHF at excessively high temperatures. Strengthening catalyst acidity to drive dehydration may trigger excessive GVL ring‐opening. Enhancing Ru hydrogenation activity to improve conversion may lower GVL selectivity through increased C−C bond hydrogenolysis. These inherent tensions mean catalyst design must achieve matching of hydrogenation sites and acid sites at the molecular level, requiring genuine integration of synthetic chemistry, surface science, and reaction engineering methodologies rather than fragmented parameter optimization.
4. Absence of standardized long‐term stability evaluation systems. Recyclability testing conditions vary enormously across the literature, rendering objective cross‐study comparison impossible. Establishing a standardized evaluation protocol encompassing true hydrothermal acidic conditions, multi‐cycle long‐duration continuous testing, quantitative metal leaching analysis, and catalyst structural integrity characterization constitutes essential foundational work for driving field consensus and accelerating industrial catalyst design.

### 4.4 Research Priorities by Development Stage

Future research on Ru‐catalyzed conversion of LA and levulinates to GVL should distinguish among three priority areas: basic research, process development, and application deployment (Figure 12). These stages address different research questions. A catalyst that aids in identifying active sites may not be suitable for continuous operation or the conversion of actual feedstocks. Data analysis based on experimental studies and artificial intelligence (AI)‐driven machine learning can advance these three areas, but their value depends on the availability of standardized and sufficiently comprehensive data.

(1) The primary focus of basic research is to identify active Ru structures under reaction conditions and determine how they control the rates of elementary reactions. A distinction should be made between systems such as isolated Ru atoms, sub‐nanometer clusters, nanoparticles, and single‐atom/nanoparticle dual active sites, rather than lumping them together into a single size‐activity relationship. Therefore, single‐atom constructs serve as a platform for elucidating the functionality of active sites. Future research should determine whether isolated Ru atoms independently activate H_2_, whether adjacent Ru atoms or particles provide surface‐active hydrogen, and how the distance between Ru and acid sites affects hydrogenation and cyclization reactions. In situ XAS, isotope experiments, transient kinetics, and analyses of the degree of rate control in microkinetics are highly useful for addressing these questions. Carefully curated data under specific reaction conditions can assist in the screening of reaction evidence descriptors and uncertainty analysis, as well as in selecting experiments capable of distinguishing between competing mechanisms. However, current literature data are not yet sufficient to support reliable predictions across different catalysts and reaction conditions.
(2) Process development requires the establishment of universal and reliable kinetic and stability testing protocols to ensure comparability across different reaction systems. Initial reaction rates should be measured after excluding gas‐liquid mass transfer, as well as external and internal mass transfer limitations. TOF values relative to Ru or active sites should be reported, along with substrate concentration, conversion range, catalyst loading, and reaction conditions. Continuous testing should report feed composition, hydrogen flow rate, continuous operating time, cumulative GVL yield calculated based on the initial Ru mass of the catalyst, Ru loss, carbon balance, spent catalyst structure, and regeneration methods. In addition to the activity of powdered catalysts, evaluations should also cover catalyst sintering, pressure drop, heat and mass transfer, support stability in aqueous reaction systems, and product separation. Extended continuous model‐substrate testing is recommended to improve comparability, but no fixed operating duration should be interpreted as an established benchmark or a criterion for industrial service life.
  New reaction modes, such as light‐assisted, photocatalytic, electrocatalytic, and magnetically induced reactions, should be evaluated using common metrics for energy and reactant inputs. Relevant information includes light or electrical energy input, a control group under matched temperature conditions, hydrogen or hydrogen donor consumption, photonic quantum efficiency or Faradaic efficiency, carbon balance, and reaction stability. Standardized reporting can also support data collation, kinetic analysis, and exploratory machine learning modeling under comparable reaction conditions.
(3) Applied research should prioritize the continuous conversion of actual biomass hydrolysates and the effects of impurities. Furfural derivatives, lignin derivatives, inorganic ions, sulfur‐containing compounds, and humic precursors may cause various problems, such as blocking ruthenium active sites, altering acid site functionality, promoting ruthenium leaching, or leading to carrier instability. Therefore, a catalyst should only proceed to the actual feed testing phase after it has maintained yield during continuous simulated feed operation and its primary deactivation mechanisms have been identified. Actual feed studies should quantify yield, Ru loss or leaching, catalyst pore blockage, structural changes, regeneration efficiency, and GVL production per unit mass of Ru. Ru recovery rate and product separation efficiency should also be included in the process evaluation. Comparisons with Ni‐, Cu‐, Co‐, and Zr‐based routes should consider requirements such as the hydrogen source, temperature, reactor configuration, catalyst lifetime, metal recovery rates, and product separation for the actual reaction. Economic evaluations of different metal catalysts should be based on measured yields, lifetime, Ru recovery rates, H_2_ or hydrogen donor consumption, and energy consumption for product separation, rather than on isolated laboratory data.

**FIGURE 12:**
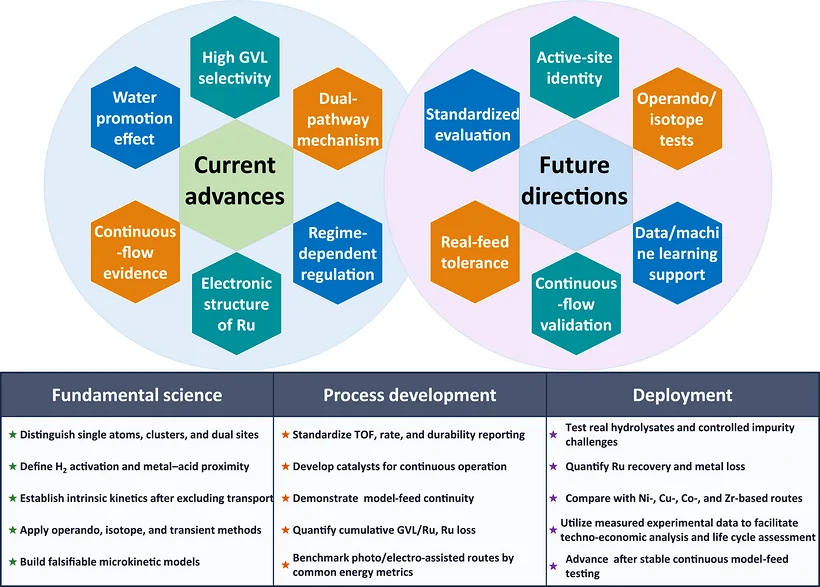
Summary of current advances and stage‐specific future priorities for Ru‐based catalysts in the conversion of LA/levulinates to GVL. Future research is organized into fundamental‐science, process‐development, and deployment priorities, with emphasis on active‐site identification, standardized evaluation, continuous‐flow validation, realistic‐feed tolerance, and data‐supported catalyst development.

Progress through each stage should follow clear criteria. Conclusions from basic research must be based on kinetic models that account for mass transfer effects and clearly identified active sites. The selection of a process scheme requires continuous simulated feed operations and the provision of comprehensive data on mass balances between substrates and products, as well as metal losses. The implementation of a process scheme must ensure that the catalyst exhibits tolerance to actual feed conditions and quantify Ru losses, catalyst regeneration, and recovery. The final process selection should depend on whether the cumulative GVL productivity per unit mass of Ru, along with other factors, is sufficient to offset the costs of the catalyst, energy input, and product separation.

Basic research, process development, and applied research can be conducted in parallel. However, basic scientific research should first identify the active sites in the working state and their intrinsic kinetic properties after eliminating mass transfer limitations (direction 1). Single‐atom and dual‐active‐site catalysts serve as platforms for elucidating H_2_ activation and the influence of metal‐acid site distances, while in situ characterizations, isotope experiments, transient kinetics, and microscopic kinetic modeling serve as experimental methods for evaluation. Subsequently, process development research should establish standardized criteria for activity and temperature performance and validate continuous simulated feed operations based on reported total operating time, cumulative GVL yield per unit mass of initial Ru, Ru loss, carbon balance, and regeneration data (direction 2). Additionally, universal metrics such as energy input, H_2_ or hydrogen donor consumption, conversion efficiency, and stability can be used to evaluate light‐induced, electro‐induced, and magnetically induced pathways. Data analysis and machine learning should remain exploratory until sufficiently comprehensive and condition‐specific data are obtained to enable experimental validation and uncertainty assessment. Industrial deployment should only proceed after performance stability under simulated feed conditions has been demonstrated and continuous operation is supported, with priority given to the economic viability of catalyst lifespan and cumulative GVL yield under conditions involving actual hydrolysates, controlled impurity effects, Ru recovery, and energy consumption for product separation (direction 3).

## Author Contributions

**Xin Ni**: writing – original draft, data curation, formal analysis. **Cailu Xiao**: formal analysis, data curation. **Gongbing Zhou**: funding acquisition, writing – review and editing, supervision, project administration. **Jingzi Zhang**: funding acquisition, writing – review and editing, project administration, supervision.

## Conflicts of Interest

The authors declare no conflicts of interest.

## Data Availability

Data sharing not applicable to this article as no datasets were generated or analysed during the current study.
